# A novel peptide‐based tau aggregation inhibitor as a potential therapeutic for Alzheimer's disease and other tauopathies

**DOI:** 10.1002/alz.14246

**Published:** 2024-10-03

**Authors:** Anthony Aggidis, George Devitt, Yongrui Zhang, Shreyasi Chatterjee, David Townsend, Nigel J. Fullwood, Eva Ruiz Ortega, Airi Tarutani, Masato Hasegawa, Amber Cooper, Philip Williamson, Ayde Mendoza‐Oliva, Marc I. Diamond, Amritpal Mudher, David Allsop

**Affiliations:** ^1^ Department of Biological Sciences University of Southampton Southampton UK; ^2^ Division of Biomedical and Life Sciences University of Lancaster Lancaster UK; ^3^ Department of Science and Technology Nottingham Trent University Nottingham UK; ^4^ Department of Chemistry University of Lancaster Lancaster University Lancaster UK; ^5^ Department of Dementia and Higher Brain Function Tokyo Metropolitan Institute of Medical Science Tokyo Japan; ^6^ Center for Alzheimer's and Neurodegenerative Diseases Peter O'Donnell Jr. Brain Institute University of Texas Southwestern Medical Center Dallas Texas USA

**Keywords:** aggregation, Alzheimer's disease, amino‐acid, dementia, *Drosophila*, drug development, inhibitor, in silico, in vitro, in vivo, melanogaster, peptide, tau, tauopathies, therapeutic, VQIINK, VQIVYK

## Abstract

**INTRODUCTION:**

As aggregation underpins Tau toxicity, aggregation inhibitor peptides may have disease‐modifying potential. They are therefore currently being designed and target either the ^306^VQIVYK^311^ aggregation‐promoting hotspot found in all Tau isoforms or the ^275^VQIINK^280^ aggregation‐promoting hotspot found in 4R isoforms. However, for any Tau aggregation inhibitor to potentially be clinically relevant for other tauopathies, it should target both hotspots to suppress aggregation of Tau isoforms, be stable, cross the blood‐brain barrier, and rescue aggregation‐dependent Tau phenotypes in vivo.

**METHODS:**

We developed a retro‐inverso, stable D‐amino peptide, RI‐AG03 [Ac‐rrrrrrrrGpkyk(ac)iqvGr‐NH2], based on the ^306^VQIVYK^311^ hotspots which exhibit these disease‐relevant attributes.

**RESULTS:**

Unlike other aggregation inhibitors, RI‐AG03 effectively suppresses aggregation of multiple Tau species containing both hotspots in vitro and in vivo, is non‐toxic, and suppresses aggregation‐dependent neurodegenerative and behavioral phenotypes.

**DISCUSSION:**

RI‐AG03 therefore meets many clinically relevant requirements for an anti‐aggregation Tau therapeutic and should be explored further for its disease‐modifying potential for Tauopathies.

**Highlights:**

- Our manuscript describes the development of a novel peptide inhibitor of Tau aggregation, a retro‐inverso, stable D‐amino peptide called RI‐AG03 that displays many clinically relevant attributes. We show its efficacy in preventing Tau aggregation in both in vitro and in vivo experimental models while being non‐toxic to cells. RI‐AG03 also rescues a biosensor cell line that stably expresses Tau repeat domains with the P301S mutation fused to Cer/Clo and rescues aggregation‐dependent phenotypes in vivo, suppressing neurodegeneration and extending lifespan.
- Collectively our data describe several properties and attributes of RI‐AG03 that make it a promising disease‐modifying candidate to explore for reducing pathogenic Tau aggregation in Tauopathies such as Alzheimer's disease. Given the real interest in reducing Tau aggregation and the potential clinical benefit of using such agents in clinical practice, RI‐AG03 should be investigated further for the treatment of Tauopathies after validation in mammalian models.
- Tau aggregation inhibitors are the obvious first choice as Tau‐based therapies as much of Tau‐mediated toxicity is aggregation dependent. Indeed, there are many research efforts focusing on this therapeutic strategy with aggregation inhibitors being designed against one of the two aggregation‐promoting hotspots of the Tau protein. To our knowledge, RI‐AG03 is the only peptide aggregation inhibitor that inhibits aggregation of Tau by targeting both aggregation‐promoting hotspot motifs simultaneously. As such, we believe that our study will have a significant impact on drug discovery efforts in this arena.

## 1 BACKGROUND

The global societal cost of dementia was estimated at US $1 trillion in 2018 and is predicted to increase to US $2 trillion by 2030.^1^ This highlights the pressing need to develop disease‐modifying treatments and in recent years, therapies that clear pathological protein aggregates are looking promising.^2^

Alzheimer's Disease (AD) as a proteopathy is characterized by accumulation of extra‐cellular plaques composed of β‐amyloid peptide (Aβ) and intra‐cellular neurofibrillary tangles (NFTs) of hyper‐phosphorylated Tau protein. Protein aggregates are damaging to cells, with smaller oligomers often regarded as the most neurotoxic forms of both Aβ and Tau.^3^ Hyperphosphorylation causes Tau to misfold and adopt rich β‐sheet content, which initiate a nucleation‐dependent aggregation process.^4^, ^5^, ^6^, ^7^ Protein monomers stack in register and parallel to each other to produce smaller soluble oligomers and ultimately larger insoluble protofilaments, forming paired helical filaments (PHFs) or straight filaments found in NFTs and other Tau inclusions, respectively.^8^, ^9^, ^10^, ^11^, ^12^

Tau has six isoforms dependent on alternative mRNA splicing from the *MAPT* gene on human chromosome 17. The absence or presence of exon 10 determines if Tau has 3 or 4 microtubule‐binding repeats.^13^ This exon encodes the second repeat, which includes aggregation hotspot ^275^VQIINK^280^, whereas the third repeat includes the other aggregation hotspot ^306^VQIVYK^311^. These hexapeptides are essential for aggregation^14^, ^15^ and are required for Tau‐dependent toxicity in animal models of Tauopathy, including the *Drosophila* model used in this study.^16^

Over the past few years, there has been great interest in Tau‐centered disease modifying therapies.^2^ These therapies include agents that compensate for loss‐of‐Tau function, including microtubule stabilizing agents Davunetide/NAPVSIPQ^17^ and Epitholone,^18^ agents that reduce Tau phosphorylation,^19^ clear Tau aggregates (through passive and active immunotherapy),^20^ reduce Tau expression using anti‐sense oligonucleotides,^21^ and molecules that reduce Tau aggregation.^22^ Several Tau aggregation inhibitors have been described and they reduce aggregation by a number of different mechanisms. Some inhibitors are “capping agents,” usually peptides that interact with and block Tau aggregation hotspots ^275^VQIINK^280^ or ^306^VQIVYK^311^.^23^, ^24^, ^25^, ^26^, ^27^, ^28^ Others are broad‐spectrum small molecules that prevent polymerization of multiple aggregate‐prone proteins, like Azure B^29^, ^30^ and other Methylene Blue derivatives,^31^ hepta‐histidine,^32^ cationic arginine‐rich peptides,^33^ and others.^34^ These inhibitors have been tested in vitro primarily with recombinant Tau fragments, in cell culture to assess toxicity and ability to reduce intracellular Tau aggregation, and to a smaller extent, in in‐vivo models of Tauopathy. Only methylene‐blue derivatives have thus far been tested in clinical trials, highlighting the conceivable therapeutic potential of Tau aggregation inhibitors, and the need to develop more, ideally Tau‐specific, anti‐aggregation agents.

We have designed peptide aggregation inhibitors to target Tau aggregation “hotspot” ^306^VQIVYK^311^.^35^ Final candidate inhibitor RI‐AG03 (Retro inverted Aggidis generation 3) suppressed aggregation of peptides containing either or both “hotspots” including the ^306^VQIVYK^311^ hexapeptide (motif found in all six isoforms), ^275^VQIINK^280^ hexapeptide (motif found in three isoforms), Tau^Δ1‐250^ (an aggregate prone truncated version of full‐length Tau), and full‐length Tau (Tau^2N4R^). It was then tested against Tau^2N4R^ seeded aggregation in mammalian cell culture and aggregation of pathological hyperphosphorylated human Tau^2N4R^ (hTau^2N4R^) in vivo in a *Drosophila* model of Tauopathy. In this model, RI‐AG03‐mediated suppression of aggregation in vivo correlated with the reduction of aggregation‐dependent phenotypes without causing toxicity.

## 2 METHODS

**Docking**: Test peptides were docked without bias onto Tau structures from the PDB using Molsoft ICM‐Pro (*version 3.8‐7*). They did not include cell penetrating sequences due to protocol limitations. Water was kept tight and His, Pro, Asn, Gln, and Cys side chains were optimized to correct orientation and H‐bond network. Standard ICM‐dock 3D grid potential maps used 0.5 Å grid spacing and docking probes were placed on the outer chains.^36^, ^37^ Ligand atom types and charges were assigned by the Merck molecular force field. “Peptide docking mode” forcefield was used to dock ligands onto Tau structures with maximum sampling effort factor.^38^, ^39^ Flexible ring sampling (2) and biased probability Monte Carlo randomly selected independent subspaces based on predefined continuous probability distribution.^40^

**Recombinant protein expression**: For Tau^Δ1‐250^, expression plasmid pRK‐172 was transformed into *E. coli* BL21(DE3) cells and expressed as described.^41^ Taniguchi‐Watanabe received 4R1N in pRK‐172 from Dr. Michel Goedert.^42^ For Tau^2N4R^, pET‐29b tau plasmid (addgene, NM_005910) was transfected into *E. coli* BL21 cells as described.^43^, ^44^

**RESEARCH IN CONTEXT**

1. **Systematic review**: The global societal cost of dementia is predicted to increase to US $2 trillion by 2030.^1^ This highlights the pressing need to develop disease‐modifying treatments. Tau aggregation inhibitors have been devised and studied for the last 20 years with several initial reports describing the promising potential of numerous small molecules which employ different mechanisms of action.^65^, ^74^, ^75^ Thus far, only methylene‐blue derivatives have been tested in clinical trials and have failed, highlighting the conceivable therapeutic potential of Tau aggregation inhibitors, and the need to develop more, ideally Tau‐specific, anti‐aggregation agents. While there is no doubt that small molecules employ different mechanisms to effectively reduce Tau aggregation, their utility as therapeutic agents are limited because their mode of action is invariably non‐specific so they invariably impact other proteins and cause unwanted side effects.^63^
2. **Interpretation**: As aggregation underpins Tau toxicity, aggregation inhibitor peptides may have disease‐modifying potential in Tauopathies such as Alzheimer's disease. They are therefore currently being designed and target either the ^306^VQIVYK^311^ aggregation‐promoting hotspot found in all Tau isoforms or the ^275^VQIINK^280^ aggregation‐promoting hotspot found in 4R isoforms. However, for any Tau aggregation inhibitor to be clinically relevant across all Tauopathies, it should target both hotspots to suppress aggregation of all Tau isoforms, be stable, cross the blood‐brain barrier, and rescue aggregation‐dependent Tau phenotypes in vivo. We describe the development of a novel peptide inhibitor of Tau aggregation, a retro‐inverso, stable D‐amino peptide called RI‐AG03, which to our knowledge is the only peptide aggregation inhibitor that inhibits aggregation of Tau by targeting both aggregation‐promoting hotspot motifs simultaneously. We show its efficacy in preventing Tau aggregation in both in vitro and in vivo experimental models while being non‐toxic to cells. RI‐AG03 also rescues aggregation‐dependent phenotypes in vivo, suppressing neurodegeneration and extending lifespan.
3. **Future directions**: Thus, we believe that our study will have a significant impact on drug‐discovery efforts in this arena, and RI‐AG03 is an excellent candidate for future exploration to ultimately treat Tauopathy patients.

**Recombinant protein purification**: For Tau^Δ1‐250^, cells were resuspended in lysis buffer (50 mM PIPES, 1 mM EGTA, 1 mM DTT, 0.5 mM PMSF, 0.5 µg/mL leupeptin, pH 6.8) and lysed via sonication. The supernatant was supplemented with 1% β‐mercaptoethanol, boiled for 5 min, and centrifuged at 27,000 × *g*. The supernatant was loaded onto a sulphopropyl sepharose column. Tau^Δ1‐250^ was eluted with purification buffer (50 mM PIPES, 1 mM EGTA, 1 mM DTT, pH 6.8) + 0.35 M NaCl. Tau^Δ1‐250^ was precipitated at 35% ammonium sulfate. Pellets were reconstituted in ddH_2_O and dialyzed against Tris (30 mM), pH 7.5. For Tau^2N4R^, Bacteria were grown at 37°C in LB broth with 20 µg/mL kanamycin to an optical density of 0.5–0.6 (600 nm absorbance). 1 mM isopropyl β‐d‐thiogalactopyranoside (IPTG) was added to induce expression for 3.5 h. Bacteria were sedimented for 20 min at 5000 × *g* and stored at −20°C overnight. Pellets were resuspended in homogenization buffer (20 mM MES, 50 mM NaCl, 1 mM MgCl2, 1 mM EGTA, 5 mM DTT, 1 mM PMSF, cOmplete™ Protease Inhibitor Cocktail, pH 6.8) and sonicated on ice. Cell homogenate was boiled at 95°C for 20 min and centrifuged at 127,000 × *g* for 45 min at 4°C. The supernatant was dialyzed against buffer A (20 mM MES, 50 mM NaCl, 1 mM MgCl2, 1 mM EGTA, 2 mM DTT, 0.1 mM PMSF pH 6.8) overnight (25 kDa cutoff, Spectra/Por) and samples were loaded onto a cation exchange column (GE Healthcare) and eluted against increasing concentrations of NaCl from buffer B (20 mM MES, 1 M NaCl, 2 mM DTT, 1 mM MgCl2, 1 mM EGTA, 0.1 mM PMSF pH 6.8). Protein was precipitated overnight at 4°C in an excess of ice‐cold methanol (1:2–1:4 volume:volume). Protein was sedimented by centrifugation at 4000 × *g* for 20 min at 4°C, methanol was decanted, and pellets were dried in a fume hood for 30 min. Pellets were resuspended in a total of 2 mL 8 M guanidine hydrochloride (Gdn HCl, Sigma) and rotated for 1 h at RT. The buffer was exchanged to PBS (10 mM Na2HPO4, 2 mM KH2PO4, 137 mM NaCl, 2.7 mM KCl, 2 mM DTT pH 7.4) using a PD‐10 desalting column (GE Healthcare) as per manufacturer's instructions. Tau protein was diluted to 20 µM, snap‐frozen in liquid nitrogen, and stored at −80°C in 1 mL aliquots.

**Synthesis of peptides**: Peptides were synthesized using standard Fmoc chemistry, employing solid phase support (Rink Amide AM Resin, GL Biochem) and Fmoc‐protected amino acids (e.g., D‐amino acids or acetylated D‐amino acids) where required. Peptides were synthesized with amino C‐termini and were capped with acetyl groups prior to cleavage. Fluorescent compounds were added as required at the N‐Terminus. Coupling of amino acids was achieved using Hexafluorophosphate Benzotriazole Tetramethyl Uronium (GL Biochem) and N,N‐Diisopropylethylamine (Fluorochem). After chain completion, RI‐AG03 was capped at the N‐terminus (Acetic anhydride, Sigma Aldrich), and fluorescently tagged RI‐AG03 was further reacted at the N‐terminus with 5(6)‐TAMRA (GL Biochem). Peptide cleavage was performed using 95% Trifluoroacetic acid (Fluorochem) with scavengers Triisopropyl silane (Alfa Aesar), Anisole (Alfa Aesar), and 1,2‐Ethanedithiol (Fluorochem) for 2 h before filtration, concentration in vacuo and freeze‐drying. Mass spectrometry was performed by MALDI‐TOF and Quadrupole Electrospray MS from LCMS. HPLC purification was performed on preparative scale (Waters 600, 0%–50% Acetonitrile in water with 0.1% TFA counterion for 120 mins at 230 nm using a Jupiter Proteo 90A 250 × 22.10 mm 16‐micron column), and then analytical scale (Kontron 420, 0%–100% Acetonitrile in water with 0.1% counterion for 30mins using a Jupiter Proteo 90A 250 × 4.6 mm 4‐micron column).

**Thioflavin‐T fluorescence (ThT)**: For kinetic experiments, aggregation mix (Tau^Δ1‐250^/peptide 20 µM, ThT 15 µM, dithiothreitol 1 mM, Tris 30 mM, and heparin 5 µM pH 7.4) was incubated at 37°C with 10 s of shaking every 10 min for 24 h. For end‐point experiments, aggregation mix (Tau^2N4R^ 20 µM, ThT 20 µM, dithiothreitol 1 mM, PBS and heparin 10 µM pH 7.4) was incubated at 37°C with constant shaking (220 rpm) for 216 h (9 days). Fluorescence was measured (λex = 442 nm, λem = 483 nm), and data were normalized. To generate a suitable ThT response, 20 µM Tau was used as a standard across experiments. The addition of inhibitor to the growth phase was controlled by adding an equivalent volume of buffer alone.

**Transmission electron microscopy (TEM)**: Tau was aggregated in similar conditions as above. Samples (10 µL) were loaded onto Formvar/Carbon 300 mesh copper grids for 3 min. The excess sample was wicked away and then negatively stained with 10 µL of filtered 2% phosphotungstic acid, pH 2, for 2 min. Grids were examined using a JEOL JEM‐1010 transmission electron microscope and were visualized and photographed at 50,000× magnification with 80.0 kV.

**Circular dichroism (CD)**: CD spectra were obtained using a Chirascan plus qCD spectrometer between 180 and 260 nm with a bandwidth of 1 nm and a path length of 2 mm.

**Enzyme stability**: RI‐AG03 (20 µM) was incubated at 37°C for 24 h with and without an equimolar concentration of Trypsin and run on SDS‐PAGE gels.

**Cell culture**: HEK‐293 cells were maintained at 37°C, 5% CO_2_ in Dulbecco's Modified Eagle Medium/Ham's F12 (DMEM/F12) at 1:1 supplemented with 10% fetal bovine serum (FBS) and 1% antibiotics (Streptomycin and Penicillin at 1:1).

**Cellular uptake**: Sterile VWR pcs Cover Glasses (13 mm) were placed into wells of a 12‐well plate. HEK‐293 cells were seeded into individual wells at 20,000 cells, supplemented with 5(6)‐carboxyfluorescein conjugated peptide inhibitors and incubated for 24 h. The media was washed off and the cells were fixed using 4% formaldehyde, washed with TBS, and mounted to microscope slides. Samples were visualized the FITC filter on a Nikon Eclipse Ti fluorescent microscope.

**Tau biosensor cells culture**: RD P301S Tau biosensor cells have been previously described,^45^ and in this case v2L cells were used. Cells were cultured in 10 cm dishes containing Dulbecco's Modified Eagle's Medium (DMEM) (Gibco) enriched with 10% FBS (HyClone), 1% penicillin/streptomycin (Gibco), and 1% GlutaMAX (Gibco) at 37°C with 5% CO2 in a humidified incubator.

**Seeding in Tau biosensor cells**: Recombinant Tau FL WT (2N4R) fibrils were prepared with heparin at 1:1 molar ratio as previously described^46^ 5 µM of tau fibrils (monomer equivalent) were incubated in PBS with peptide RI‐AG03 or scramble (0–500 µM) at 300 rpm agitation at 4°C for 16 h. The reaction was stopped by flash freezing in liquid nitrogen and stored at –20°C. Tau biosensor cells were plated at a density of 30,000 cells in 120 µL of media per well in a 96‐well plate. After 18 h, using Lipofectamine 2000 cells were transduced with 10 nM of treated fibrils that had been briefly sonicated for 30 s at 65 Amp (QSonica). Lipofectamine complexes were formed in 30 µL consisting of 14.17 µL Opti‐MEM (Gibco), 15 µL of treated fibrils diluted in PBS, and 0.83 µL lipofectamine 2000 (Invitrogen), bringing the total volume to 150 µL. Preparations were incubated at room temperature for 30 min before being added. Cells were incubated with treated fibrils for 24 h. After incubation, cells were collected with 0.05% trypsin, quenched with media, fixed in 2% paraformaldehyde for 10 min, and resuspended in PBS. Aggregates in Tau biosensors (10,000 minimum) were quantified by FRET flow cytometry in a LSRFortessa SORP (BD Biosciences). To detect the cerulean signal, cells were excited with a 405 nm laser, and fluorescence was captured at 405/50 nm. For clover signal, cells were excited with a 488 nm laser and fluorescence was detected at 525/50 nm. Flow cytometry data were analyzed using a gating strategy previously described.^45^, ^47^ The percentage of cells with positive FRET signal were quantified. Each condition was analyzed in quadruplicate. Data analysis was performed using FlowJo v10 software (Treestar Inc.) for Mac.

***Drosophila* stocks**: *Drosophila melanogaster* expressing either the retinal photoreceptor‐specific *GMR*‐GAL4 driver or pan‐neuronal driver *ElavC155*‐GAL4, *UAS‐*Tau^2N4R^, and Oregon‐R (WT) flies were obtained from Bloomington Stock Centre, Indiana.

***Drosophila* eye experiments**: *UAS*‐Tau^2N4R^ flies (BDSC_51363) were recombined to *GMR*‐GAL4 (BDSC_8605) driver to generate stable *GMR*‐hTau^2N4R^ lines (BDSC_51361). *GMR*‐GAL4 was used as the “healthy” control. Progeny was reared on standard fly food supplemented with RI‐AG03; 0.08, 0.8, 20, and 40 µM at 25°C. As controls, Tau, and driver‐alone flies were incubated without the inhibitor. Freshly eclosed flies were monitored daily using a light microscope and 1‐day‐old live flies were imaged (*n* = 5/treatment) and processed on ImageJ to measure eye width. Ommaditial disorderliness quantification was done using Flynotyper ImageJ plugin.^48^

**Quantification of rough eye phenotype**: Quantitative analysis of the total distance ommatidial disorderliness index of all stable ommatidia [Odld] was performed using the ImageJ plugin “Flynotyper.”^48^ Analysis was performed using images taken by light microscope, and 150 ommatidia were considered and ranked by stability and distance to the center. Between four and five animals per genotype were analyzed. 

**Scanning Electron Microscopy (SEM)**: Flies were euthanized, fixed in 2.5% glutaraldehyde in PBS, and had three 5‐min washes in PBS. Samples were dehydrated for 30‐min at each ethanol concentration: 50%, 70%, 80%, 90%, and 100%. Samples were transferred to hexamethyldisilazane for two 30‐min changes, after which hexamethyldisilazane was sublimated overnight. Samples were mounted onto JEOL SEM stubs before sputter coating with gold for 4‐min on an Edwards 150A sputter coater. Samples were examined using a JEOL 5600 SEM at 130× magnification with 30.0 kV.

***Drosophila* survival assays**: *Elav*‐GAL4 (BDSC_458) female flies were crossed with male *UAS*‐Tau^2N4R^ flies or with gender‐matched Oregon‐R (WT) flies. Survival of 10 cohorts of 10 male flies expressing hTau^2N4R^ pan‐neurally and reared on a diet containing 0.08 or 0.8 µM RI‐AG03 was assessed as previously described.^49^

**Solubility assay to enriching for insoluble Tau (NS) for atomic force microscopy (AFM)**: *Elav*‐GAL4 (BDSC_458) adult flies (*n* = 20) pan‐neurally overexpressing full‐length human Tau (*UAS‐*Tau^2N4R^) were treated with 0.08 mM RI‐AG03 over 6 weeks. Decapitated fly heads were homogenized in TBS/sucrose buffer (50 mM Tris‐HCl pH 7.4, 175 mM NaCl, 1 M sucrose, 5 mM EDTA and protease inhibitor cocktail). Samples were spun for 2 min at 1000 *g*, retaining the supernatant, which was then spun at 100,000 *g* for 30 min at 4°C. The supernatant included the aqueous soluble fraction and monomeric Tau “NS1.” The pellet was re‐suspended at ∼20°C–25°C in 5% SDS/TBS buffer (50 mM Tris‐HCl pH 7.4, 175 mM NaCl, 5% SDS, and protease inhibitor cocktail) and spun at 100,000 *g* for 30 at 25°C. The pellet “NP1” was washed three times with water to remove the remaining SDS from the fractionation and re‐suspended in 1X PBS. A solid glass bead was added to each sample and incubated overnight in a rotor at 4°C.

**AFM**: NP1 (5 µL) was diluted in 1X PBS and 25 µL of the sample was placed in a freshly cleaved 10 mm mica disc. These were incubated at ∼20°C–25°C for 5 min. Samples were rinsed four times with ultrapure water and dried with compressed air. Samples were imaged in the air with a digital multimode Nanoscope IV AFM operating in tapping mode with an aluminum‐coated non‐contact/Tapping mode probe (resonance frequency 320 kHz and force constant 42 N/m). The images are representative of the sample and were taken at random points on the sample with a scan rate of 1–2 Hz. The acquired images were processed by WSXM software. Quantification of aggregates was carried out using ImageJ software.

**Statistics and reproducibility**: Data were collected in ≥triplicate and expressed as mean ± standard error of the mean. ThT data were analyzed using one‐factor repeated‐measures analysis of variance (ANOVA) with Tukey post hoc testing through IBM SPSS Statistics 23. This test analyzed outcomes related to the treatment effects of peptide inhibitors against Tau aggregation at multiple time points, controlling for individual variability and ensuring that changes observed were significant. For the *Drosophila* survival data, a Kaplan–Meier survival curve was plotted, and a Log‐rank (Mantel–Cox) test was performed on the data using GraphPad Prism software^49^ This test analyzed survival outcomes by comparing survival distribution between groups, without assuming a specific distribution for survival times. AFM data were analyzed by unpaired *t*‐test with Welch's correction as we made the assumption, from previous similar studies, that the two populations (Tau enriched preps isolated from flies fed or not fed RI‐AG03) had the same variance.

## 3 RESULTS

### 3.1 Designing aggregation inhibitors to target Tau's aggregation hotspots

Previously we developed aggregation inhibitors by targeting aggregation hotspots on Aβ and amylin,^50^, ^51^ and now employ a similar approach to design Tau aggregation inhibitors. In silico investigations using Aggrescan and Camsol confirmed target suitability in Tau and peptide inhibitor design through an informed guidance approach. Aggrescan predicted the aggregation propensity of experimental peptides by comparing amino acid sequences to known hot spot sequences from 57 amyloidogenic proteins.^52^ Camsol predicted the intrinsic solubility profile of experimental peptides^53^, ^54^ by evaluating amino acid hydrophobicity, aggregation propensity, charge, and secondary structure. Values >1 suggest high solubility, whereas <1 suggests poor solubility.^55^ These algorithms highlighted ^306^VQIVYK^311^ as the region in Tau most prone to aggregation and with the lowest intrinsic solubility (Figures S1 and S2).

Hexapeptide residues were optimized in silico to reduce aggregation propensity, so they do not self‐associate into β‐sheets, while also maintaining target specificity. Table S3 highlights residue optimizations on VQIxYK and VQxVYK, respectively, where “x” was systematically replaced with all possible amino acid residues. Lysine was confirmed as the optimum replacement in both VQxKYK and VQIxYK. It reduced aggregation propensity just below the aggregation threshold, suggesting retention of target affinity (Figure S4). Histidine replacement was rejected as its intrinsic insolubility matched that of ^306^VQIVYK^311^. VQIKYK was preferred as it retained some intrinsic insolubility, unlike VQKVYK (Figure S5). Acetylation of lysine neutralized the positive charge and increased its size, thereby enhancing stable binding to Tau and promoting steric hindrance to encourage an alternate fold, while retaining docking propensity for glutamine.^56^, ^57^

**FIGURE 1:**
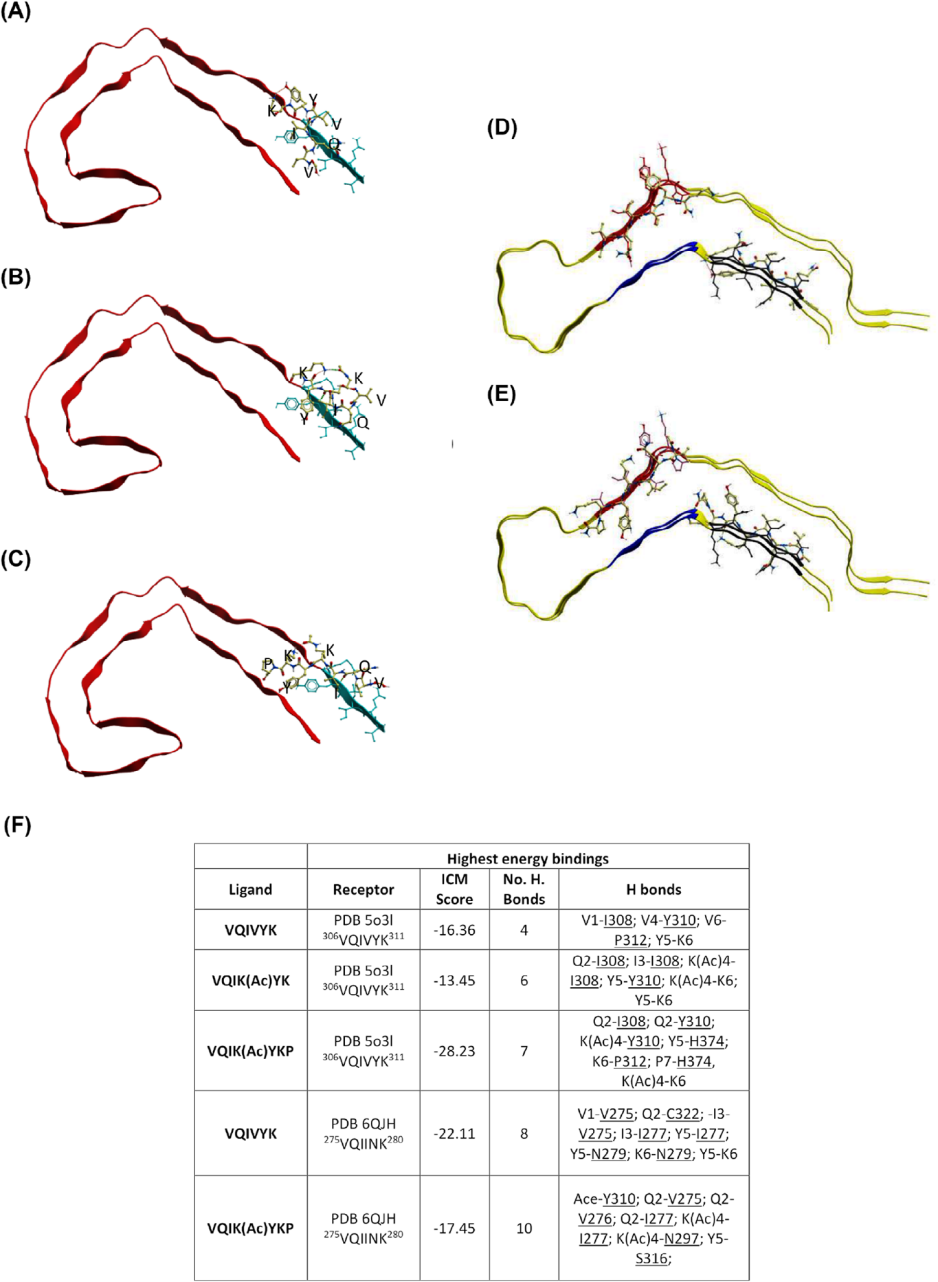
VQIK(Ac)YKP peptides dock to VQIVYK. Experimental peptides docked in parallel to the superior portion of PDB 5o3l 2N4R PHF (A–C) demonstrate preferential binding to ^306^VQIVYK^311^, whereas experimental peptides docked to PDB 6QJH 2N4R Tau snake filament (D–E) bind to both ^306^VQIVYK^311^ and ^275^VQIINK^280^. (A) Ac‐VQIVYK‐NH2; (B) AC‐VQIK(Ac)YK‐NH2; (C) Ac‐VQIK(Ac)YKP‐NH2, notice the peptide extending to interact with the parallel β‐sheet of ^306^VQIVYK^311^. (D) Ac‐VQIVYK‐NH2 binding in parallel to the filament; (E) Ac‐VQIK(Ac)YKP‐NH2 binding in anti‐parallel to the filament at ^306^VQIVYK^311^ position, and in parallel at the ^275^VQIINK^280^ position. (F) Summary table of the highest computationally calculated energy values describing the docked compounds to PDB‐5o3l and PDB‐5QJH (emphasis on VQIINK). Lower scores indicate more powerful interactions. ICM score of <–32 indicates a strong binding.

Experimental peptides were computationally docked to available human Tau Protein Data Bank (PDB) structures to predict if they would bind to their target region in vivo. Figure 1A–C summarizes the binding sites of peptides docking to human Tau^306‐378^ isolated from the brain (PDB‐5o3l). Figure 1A–E demonstrates that control peptide [VQIVYK] binds in parallel in PDB‐5o3l to ^306^VQIVYK^311^ and to^275^VQIINK^280^ in PDB‐5QJH. Figure 1F highlights the average energy interactions (*n* = 3). Based on the ICM score [VQIK(Ac)YKP] binds with greater intensity to ^306^VQIVYK^311^ in both PDB structures, however in Figure 1D–E (PDF‐5QJH) it conversely binds in anti‐parallel to ^306^VQIVYK^311^ and in parallel with similar intensity to [VQIVYK] in ^275^VQIINK^280^. Table S6 shows that [VQIVYK] and [VQIK(Ac)YKP] docked to multiple recombinant Tau structures from the PDB with similar intensity. Most noteworthy was that [VQIK(Ac)YKP] bound almost twice as strongly to PDB‐5o3l^8^ than native [VQIVYK] did. For reference, both heparin and ThT bind to Tau very strongly with ICM scores of –34.73 and –28.45. Docking experiments demonstrated that acetyl lysine, k(Ac), in the inhibitor, interacts with Tau at I308, Y310, I277, N297 and with itself on K6.

Our approach for designing experimental peptides is in line with other strategies that have used either phage display selection procedures^24^, ^58^ or structure‐based design approaches to select peptides that target aggregation‐driving hotspots on Tau.^26^ These strategies invariably targeted either the ^275^VQIINK^280^ or the ^306^VQIVYK^311^ motifs, also known as PHF6* and PHF6, respectively. However, to our knowledge, none of the Tau aggregation peptides described thus far target *both* hotspots. Though designed against ^306^VQIVYK^311^, our docking data predict that our experimental peptides can also bind ^275^VQIINK^280^ which gave us confidence that an optimized lead compound from these peptides would potentially inhibit aggregation by interacting with both aggregation hotspots. Thus, these peptides were experimentally interrogated to identify a lead peptide.

### 3.2 Selection of the lead peptide RI‐AG03

**FIGURE 2:**
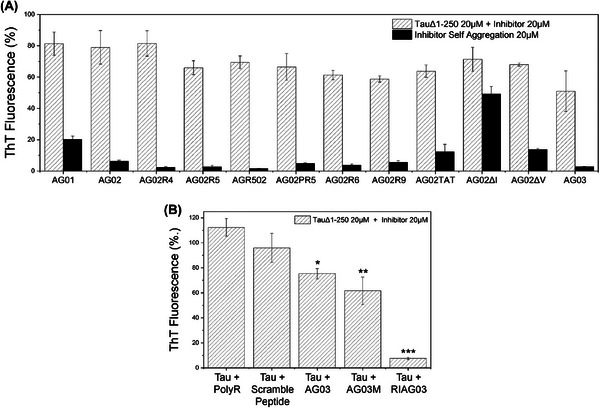
AG03 utilizes the VQIK(Ac)YKP recognition sequence. Aggregation end‐point measurements using ThT fluorescence after 24 h from 20 µM Tau^Δ1‐250^ and/or 20 µM peptide inhibitor in the presence of 30 mM Tris buffer, 1 mM DTT, 15 µM ThT, 5 µM heparin (pH 7.4). (A) Aggregation of Tau^Δ1‐250^ with different peptide inhibitors in the presence of heparin (white, hatched) and attempted self‐assembly of inhibitors without the presence of Tau (black). Key: AG01 [RG‐VQIINK‐GR], AG02 [RG‐VQIVYK‐GR], AG02R4 [RRG‐VQIVYK‐GRR], AG02R5 [RG‐VQIVYK‐GRRRR], AGR502 [RRRRG‐VQIVYK‐GR], AG02PR5 [RG‐VQIVYKP‐GRRRR], AG02R9 [RG‐VQIVYK‐GRRRRRRRR], AG02TAT [RG‐VQIVYKGRYGRKKRRQRRR], AG02ΔI [RG‐VQK(Ac)VYK‐GR], AG02ΔV [RG‐VQIK(Ac)YK‐GR], AG03 [RG‐VQIK(Ac)YKP‐GRRRRRRRR]. (B) Aggregation of Tau^Δ1‐250^ in the presence of heparin with either octa‐arginine, scrambled AG03 peptide, AG03, N‐methylated AG03, or retro‐inverso AG03. Experiments were conducted in triplicate and error bars were reported as standard deviation. Statistical analysis: *p* > 0.05, *p ≤ 0.05, **p ≤ 0.01, ***p ≤ 0.001.

Based on the computational docking predictions, a range of peptides (Table S7) were synthesized and tested for their ability to self‐aggregate (Figure 2A, black bars) and to inhibit heparin‐induced aggregation of Tau^Δ1‐250^ in vitro (Figure 2A, hashed bars). Experiments were primarily conducted using Tau^Δ1‐250^ due to its rapid aggregation into filaments (hours) in comparison to Tau^2N4R^ (days). Each peptide has Ac‐RG….GR‐NH_2_ flanking sequences to aid solubility, a strategy employed in our previous work^50^, ^51^, ^59^, ^60^

As described here and illustrated in Figure 2A, the suitability of these different peptides as aggregation inhibitors was determined by considering several factors. Peptide AG01 [Ac‐RG‐VQIINK‐GR‐NH2] self‐aggregated, so ^306^VQIVYK^311^ based peptides (AG02) were prioritized for development. Furthermore, this motif is present in all Tau isoforms. Increasing solubility of AG02 by the addition of three consecutive arginine residues (AG02R5)‐[Ac‐RG‐VQIVYK‐GRRRR] inhibited aggregation by an additional 20%, presumably through charge effects and steric hindrance. The termini that poly‐Arginine chains were located on did not affect peptide inhibitory action between AGR502 and AG02R5. Eight consecutive Arginine residues (AG02R9)‐[Ac‐RG‐VQIVYK‐GRRRRRRRR] however, reduced aggregation by an additional 10%.

Tau aggregates intracellularly so inhibitors need the capability for cell and brain penetration.^61^ The TAT cell‐penetrating sequence was also explored alongside octa‐arginine. Inclusion of TAT (AG02TAT)‐[Ac‐RG‐VQIVYK‐GRYGRKKRRQRRR‐NH2] resulted in no significant additional inhibitory benefits compared to AG02 or AG02R5. However, AG02TAT did exhibit greater self‐association into β‐sheets than all other AG02‐derived peptides so octa‐arginine was preferred. AG02PR5 was designed to mimic the native Tau ^306^VQIVYKP^312^ sequence, but no additional benefit was observed. Another peptide, AG02∆I [Ac‐RG‐VQK(Ac)VYK‐GR‐NH2], demonstrated self‐aggregation ability, agreeing with our docking predictions so was not explored further. Similarly, AG02∆V [Ac‐RG‐VQIK(Ac)YK‐GR‐NH2] was tested but demonstrated a tendency for self‐aggregation, though to a smaller extent.

Based on these data, a new peptide was created which had all the characteristics required for inhibiting Tau aggregation but with minimal self‐aggregation. This peptide was called AG03. Its sequence had the 8 poly‐Arginine chains known to enhance aggregation inhibition, as well as Proline at the end, in line with the native ^306^VQIVYK^311^ sequence. Additionally, the second Valine was replaced with Acetyl Lysine to further suppress its ability to self‐aggregate by enhancing steric hindrance. This AG03 peptide [Ac‐RG‐VQIK(Ac)YKP‐GRRRRRRRRR displayed minimal self‐aggregation and yet inhibited aggregation more than any other AG01 or AG02‐derived peptides tested (∼53% inhibition) (Figure 2A compares AG03 to AG02 and AG01). Thus, AG03 was selected as the “lead” inhibitor for further development.

AG03 needed to be proteolytically stable so methylation and retro‐inversion, attributes that confer protection against enzymatic degradation, were introduced and an N‐methylated (AG03M) [Ac‐RG‐V(m)QI(m)K(Ac)Y(m)KP(m)‐GRRRRRRRR‐NH2] and a retro‐inverted version peptide (RI‐AG03) [RI‐AG03 = Ac‐rrrrrrrG‐pkyk(ac)iqv‐Gr‐NH2] were tested against Tau aggregation. AG03M showed no improvement over AG03, but RI‐AG03 inhibited Tau aggregation by ∼94% at the equimolar concentration (Figure 2B). As a retro‐inverted D‐amino peptide, RI‐AG03 is proteolytically stable due to its altered stereochemistry and inverted backbone that interferes with enzyme‐substrate recognition (Figure S8). D‐Amino acid peptides are favorable as therapeutic agents as they can be administered orally, have been shown to cross the blood‐brain barrier and exhibit increased bioavailability to rescue Aβ‐dependent phenotypes in other models of AD.^62^ Poly‐arginine [RRRRRRRR] and scrambled AG03 [Ac‐RG‐QPKIK(Ac)YV‐GRRRRRRRR] control peptides which do not include the binding region had no effect on aggregation, highlighting the importance of the specific amino acid recognition sequence of RI‐AG03 in its mechanism of action, rather than simple charge effects.

### 3.3 RI‐AG03 inhibits aggregation of multiple human tau species in vitro

**FIGURE 3:**
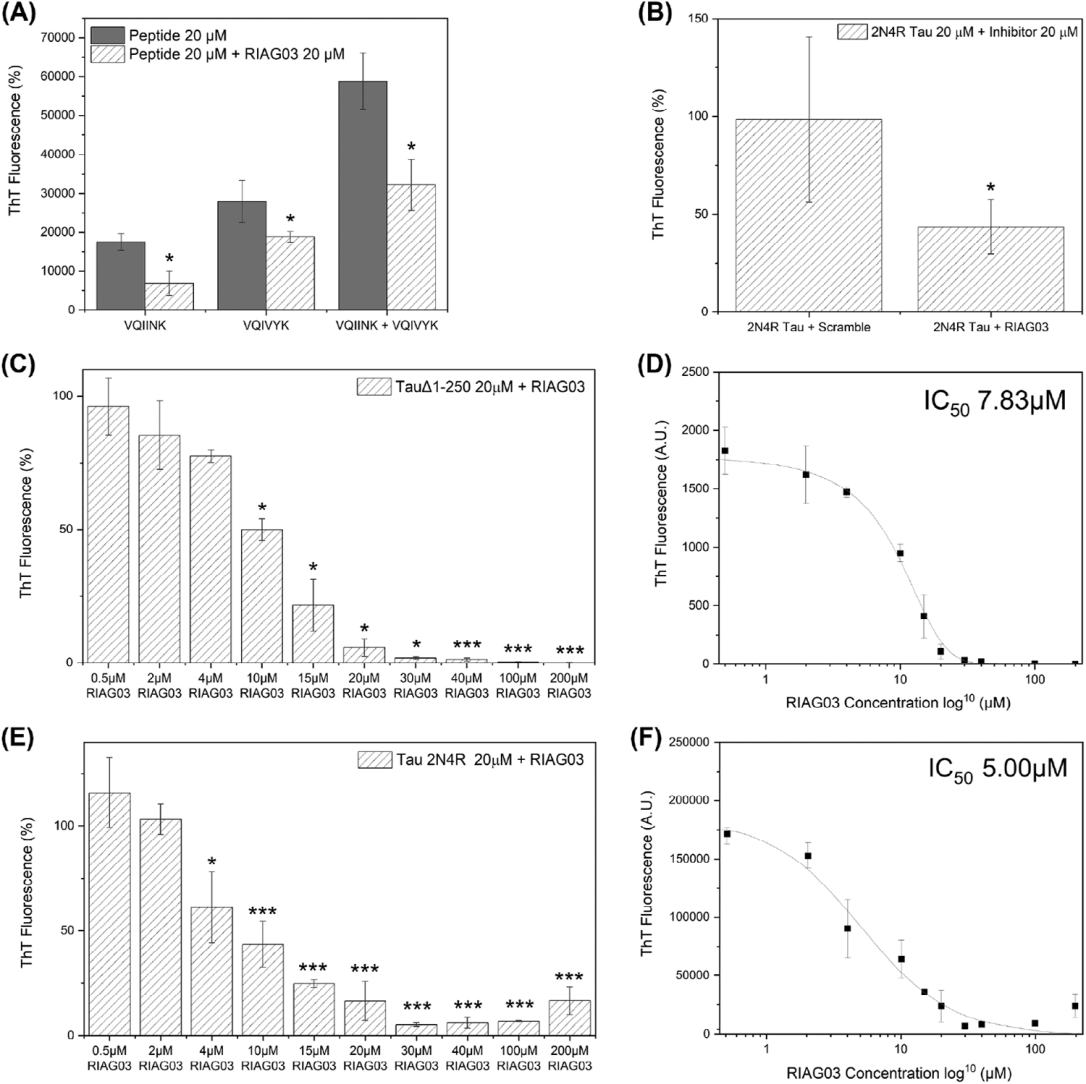
Lead peptide RI‐AG03 dose‐response and target specificity. (A) Aggregation end‐point measurements using ThT fluorescence after 24 h from 20 µM VQIINK, 20 µM VQIVYK, or a combination of both, in the presence of 30 mM Tris buffer, 1 mM DTT, 15 µM ThT, 5 µM heparin (pH 7.4), and in the presence (hatched gray bars) or absence (solid gray bars) of 20 µM RI‐AG03. (B) Aggregation end‐point measurements using ThT fluorescence after 216 h (9 days) from Tau^2N4R^ in the presence of PBS buffer, 1 mM DTT, 20 µM ThT, 10 µM heparin (pH 7.4), and 20 µM RI‐AG03 or 20 µM Scramble peptide. (C) Concentration‐dependent inhibition of by Tau^Δ1‐250^ by RI‐AG03. Aggregation end‐point measurements using ThT fluorescence after 24 h from 20 µM Tau^Δ1‐250^ in the presence of 30 mM Tris buffer, 1 mM DTT, 15 µM ThT, 5 µM heparin (pH 7.4), and RI‐AG03 in a concentration range of 0.5–200 µM. (D) log10 scatter graph of Tau^Δ1‐250^ inhibition by RI‐AG03 employing a curve fitting algorithm to calculate the IC_50_ (the concentration of RI‐AG03 required for 50% inhibition of aggregation) at 7.83 µM. (E) Concentration‐dependent inhibition of by Tau^2N4R^ by RI‐AG03. Aggregation end‐point measurements using ThT fluorescence after 216 h (9 days) of Tau^2N4R^ in the presence of PBS buffer, 1 mM DTT, 20 µM ThT, 10 µM heparin (pH 7.4), and RI‐AG03 in a concentration range of 0.5–200 µM. (F) log10 scatter graph of Tau^2N4R^ inhibition by RI‐AG03 employing a curve fitting algorithm to calculate the IC_50_ at 5 µM. Experiments were conducted in triplicate and error bars were reported as standard deviation. One factor repeated measures ANOVA + Tukey post hoc statistical analysis: *p* > 0.05, *p ≤ 0.05, **p ≤ 0.01, ***p ≤ 0.001.

Though RI‐AG03 suppresses Tau aggregation by interacting with the ^306^VQIVYK^311^ sequence on the tau protein, it would be more efficacious as an aggregation inhibitor if it could also interact with the ^275^VQIINK^280^ sequence on Tau as this is also believed to promote Tau aggregation.^26^ Encouraged by our in silico predictions that RI‐AG03 should bind to both sequences, we tested RI‐AG03's effect on the aggregation of recombinant [VQIINK] and [VQIVYK] peptides. As predicted, aggregation of both these spontaneously aggregating peptides, individually or in combination, was dramatically reduced by equimolar doses of RI‐AG03 (Figure 3A). To our knowledge, no other Tau aggregation inhibitor displays activity against both hotspots. This prompted further investigation into the effect of RI‐AG03 on other pathologically relevant Tau species like aggregate prone Tau^Δ1‐250^ peptide and Tau^2N4R^ which contain both these aggregation hotspots. As was the case for Tau^Δ1‐250^ (Figure 2B), scrambled peptide did not suppress aggregation of Tau^2N4R^ (Figure 3B), implying that suppression of Tau^2N4R^ aggregation is also directed by RI‐AG03 interaction with the aggregate‐prone domains in a sequence‐specific manner. RI‐AG03 does not self‐associate into β‐sheets within 216 h (9 days) of aggregation required for the formation of Tau^2N4R^ filaments (Figure S9). RI‐AG03 potently suppresses aggregation of both these recombinant Tau species in a dose‐dependent manner, with comparable efficacy as evidenced by similar doses of RI‐AG03 required to inhibit aggregation by 50% (IC_50_ for Tau^Δ1‐250^ = 7.83 µM Figure 3C/D; IC_50_ for Tau^2N4R^ = 5 µM Figure 3E/F).

### 3.4 What is the mechanism by which RI‐AG03 inhibits the aggregation of Tau?

As reviewed in Ref.^63^ there are multiple modes of action for Tau aggregation inhibitors.

1. M1: Direct binding of epitopes on tau protein, capping Tau in an “interaction‐incompetent” conformation, thus suppressing aggregation into oligomers or fibrils.^23^, ^25^, ^26^
2. M2: Aggregation‐hot spot mediated interactions forming stabilized off‐pathway intermediates at the expense of long fibrils.^24^
3. M3: Disaggregating or disrupting existing aggregates.^64^

For M2 and M3, it is not always clear whether suppression of Tau polymerization is accompanied by a reduction in β‐sheet structure in the alternative non‐fibrillar Tau species formed, and whether they are still amyloidogenic and therefore toxic.

**FIGURE 4:**
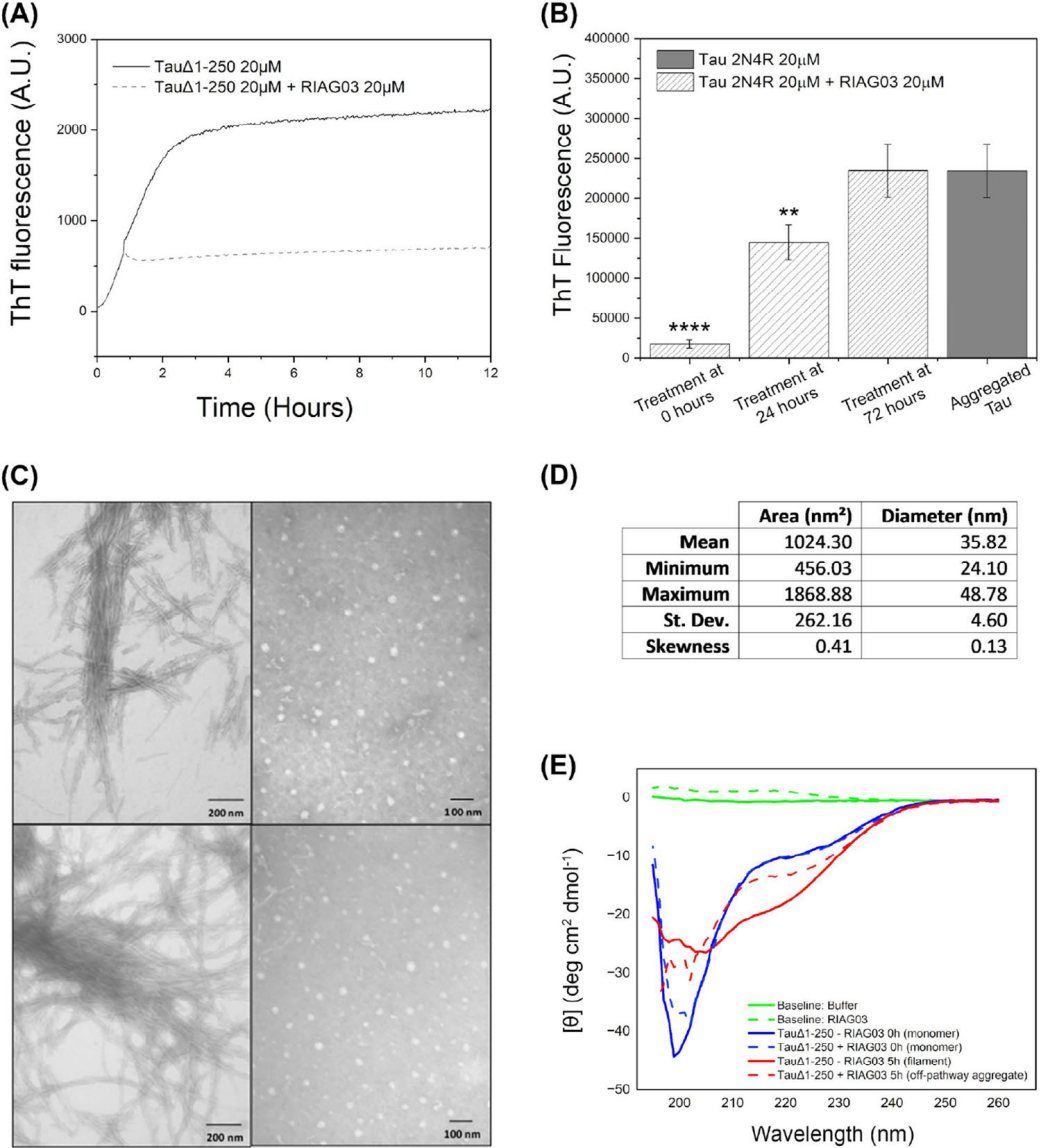
Lead peptide RI‐AG03 suppresses Tau β‐sheet content and forms of off‐pathway structures. (A) Aggregation kinetics measurements using ThT fluorescence from 20 µM Tau^Δ1‐250^ in the presence of 30 mM Tris buffer, 1 mM DTT, 15 µM ThT, and 5 µM heparin (pH 7.4), for 12 h in the absence of RI‐AG03 (solid gray line) or with the addition of 20 µM RI‐AG03 1 h after aggregation was initiated (hatched gray line). (B) Aggregation end‐point measurements using ThT fluorescence after 216 h (9 days) from 20 µM Tau^2N4R^ in the presence of PBS buffer, 1 mM DTT, 20 µM ThT, and 10 µM heparin (pH 7.4). 20 µM RI‐AG03 was added after 0, 24, and 72 h, of Tau aggregation (hatched gray bars), or not at all (solid gray bar representing end‐point aggregation of Tau^2N4R^ at 9 days). Note that Tau^2N4R^ aggregation plateaued at ∼72 h. ThT fluorescence was recorded after 216 h (9 days) for all conditions. One factor repeated measures ANOVA + Tukey post hoc statistical analysis: *p* > 0.05, *p ≤ 0.05, **p ≤ 0.01, ***p ≤ 0.001. (C) Negative stain TEM images using a Joel JEM‐1010 after the aggregation of 20 µM Tau^Δ1‐250^ for 24 h, in the absence of RI‐AG03 (left), and in the presence of 20 µM RI‐AG03 (right). (D) The area and diameter of 200 large spherical species formed in the presence of 20 µM RI‐AG03 and recorded by TEM were quantified using iTEM software. (E) Circular dichroism data depicting secondary structural changes after 20 µM Tau^Δ1‐250^ aggregation in the presence and absence of 20 µM RI‐AG03, including the baseline buffer spectrum (green solid line), and RI‐AG03 alone in buffer (green dashed line), monomeric Tau^Δ1‐250^ (blue solid line) and Tau^Δ1‐250^ aggregated for 5 h (red solid line), monomeric Tau^Δ1‐250^ in the presence of RI‐AG03 (blue dashed line) and Tau^Δ1‐250^ aggregated for 5 h in the presence of RI‐AG03 (dashed red line).

A number of methods were used to unravel which of these three aggregation inhibition mechanisms is employed by RI‐AG03, and we conclude that RI‐AG03 employs M2 as its mode of action. Our data with the scrambled peptide (Figure 2B/3B) confirm that whatever RI‐AG03's mode of action, it requires directed interaction with the ^306^VQIVYK^311^/^275^VQIINK^280^ hotspots. In an assay where RI‐AG03 was added at different time points after aggregation was initiated, RI‐AG03 demonstrated efficacy at inhibiting fibril elongation when added 1 h into the growth phase of aggregation and halted any further aggregation of Tau^Δ1‐250^ or Tau^2N4R^ (Figure 4A/B). These data rule out M3, disaggregation, as the mechanism of action for RI‐AG03 because, if it was actively disrupting preformed fibrils, there would be a reduction in ThT signal when it was added at either the 24 or 72 h timepoint where fibrils had already formed.

To find out which of the other two mechanisms of inhibition RI‐AG03 was employing, TEM was used to study the aggregation of Tau^Δ1‐250^ alone and in the presence of the inhibitor RI‐AG03. When Tau^Δ1‐250^ was incubated with heparin for 24 h, insoluble fibrils were observed (Figure 4C left side panel). However, in the presence of equimolar RI‐AG03 these fibrils were not observed, and only large spherical structures, akin to large amorphous aggregates (Figure 4C right side panel), with mean diameter ∼36 nm, were present (Figure 4D). This excludes M1, capping monomers, as off‐pathway structures are formed at the expense of longer fibrils. If RI‐AG03 caps the aggregation hotspots, it does so to stabilize the formation of off‐pathway oligomers/aggregates, perhaps like other inhibitors, that is, cyanine,^63^, ^65^ methylene blue,^66^ and peptide MMD3.^24^ However, some of these inhibitors, such as cyanine, mediate off‐pathway aggregate formation only in the presence of the aggregation inducer.^65^ To rule out similar dependence of RI‐AG03 on heparin in mediating suppression of Tau aggregation, RI‐AG03's ability to suppress seeded aggregation, induced by incubating Tau monomers with preformed Tau^Δ1‐250^ filaments was tested. As depicted in Figure S10,S RI‐AG03 does not require heparin to suppress aggregation as it demonstrated efficacy at inhibiting seeded Tau^Δ1‐250^ aggregation in the absence of heparin. This also confirms that inhibition of Tau aggregation was not caused by the inhibitor's positive charges sequestering heparin.

Tau filament formation is expected to coincide with a reduction in disordered structure and an increase in β‐sheet content, even as the intermediary on‐pathway oligomers form.^67^ In the presence of ThT, Tau aggregation is expected to coincide with an increase in fluorescence emission maximum at ∼485 nm upon binding to β‐sheets, even.^68^ However, the off‐pathway Tau aggregates that we have previously described have reduced β‐sheet content.^69^ To study the β‐sheet content of the large amorphous aggregates formed in the presence of RI‐AG03, samples were analyzed using time‐resolved circular dichroism (CD) to investigate spectra changes indicative of secondary structure in the presence of RI‐AG03 over 5 h (Tau^Δ1‐250^ aggregation plateaus at ∼5 h) (Figure 4E). The negative transition at 195 nm is characteristic of random coil structures being present within the Tau^Δ^ ^1‐250^ (blue). The reduction in the size of this negative transition, suggests that in the absence of RI‐AG03 (red), there is a reduction in the random coil content of the Tau^Δ^ ^1‐250^. Although challenging to deconvolute the increase in the negative ellipticity between ∼205 and 235 nm is consistent with the appearance of secondary structural elements including β‐strands. Upon the addition of RI‐AG03, these changes are significantly suppressed, with the smaller decrease in the negative transition at 195 nm, suggesting the persistence of random coil structures, with a smaller increase in the negative ellipticity between ∼205 and 235 nm indicating the formation of fewer secondary structural elements. These data suggest that the presence of RI‐AG03 reduces the conversion of disordered regions within Tau, to secondary structural elements including β‐strands. This reduction in β‐sheet content is corroborated by congo‐red staining, where the strong apple‐green birefringence observed for the Tau^Δ1‐250^ fibrils was eliminated when the Tau^Δ1‐250^ was incubated in the presence of RI‐AG03 (Figure S11).

Taken together, multiple lines of evidence from disparate findings in our manuscript lead us to conclude that our inhibitor acts via mechanism M2 and not M3: Our data demonstrate that the RI‐AG03 effectively inhibits aggregation: (1) Before the aggregation process (Figure 3) when Tau is in its native monomeric state, and (2) during the aggregation process (Figure 4) when there are different species present including Tau oligomers.^43^ For the end‐point assays in Figure 4B, we used ThT fluorescence as a measure of Tau^2N4R^ aggregation in the presence or absence of RI‐AG03 after 9 days. When RI‐AG03 is added to the reaction mixture after 24 h, there is a reduction of ThT signal after 9 days in comparison to the negative control, suggesting that RI‐AG03 is preventing further aggregation of monomeric Tau between 24 h and 9 days. However, when RI‐AG03 is added to the reaction mixture after 72 h, there is no reduction of ThT signal after 9 days in comparison to the negative control since the aggregation stops after 72 h and there is no further aggregation between 72 h and 9 days. If RI‐AG03 worked through the disaggregation of fibrils (M3), we would expect to see a reduction in ThT signal in this experiment. Instead, this suggests that the aggregation process is complete after 3 days and that RI‐AG03 does not reverse this process, ruling out mechanism 3 (M3). Furthermore, our CD and TEM data in Figure 4 align with M2. When RI‐AG03 is combined with monomeric Tau prior to the aggregation process, we see the formation of “off‐pathway” aggregated protein species, as observed previously by the authors.^69^

Thus, the data presented so far collectively imply that RI‐AG03 suppresses Tau aggregation by targeting the ^306^VQIVYK^311^ and the ^275^VQIINK^280^ sites to alter the aggregation pathway and encourage off‐pathway formation of large amorphous aggregates with reduced β‐sheet content instead of on‐pathway soluble oligomers that ultimately form amyloidogenic protofibrils and larger fibrils. As the toxicity of all pathogenic Tau species, whether soluble oligomers or fibrils is believed to relate to their β‐sheet content,^70^ we hypothesize that the large amorphous Tau aggregates formed in the presence of RI‐AG03 are not toxic. Indeed, we have previously described such large amorphous Tau aggregates emerging in conditions of reduced toxicity where Tau pathology was ameliorated by decreasing phosphorylation.^69^

### 3.5 RI‐AG03 is cell penetrant and suppresses Tau aggregation in vivo

**FIGURE 5:**
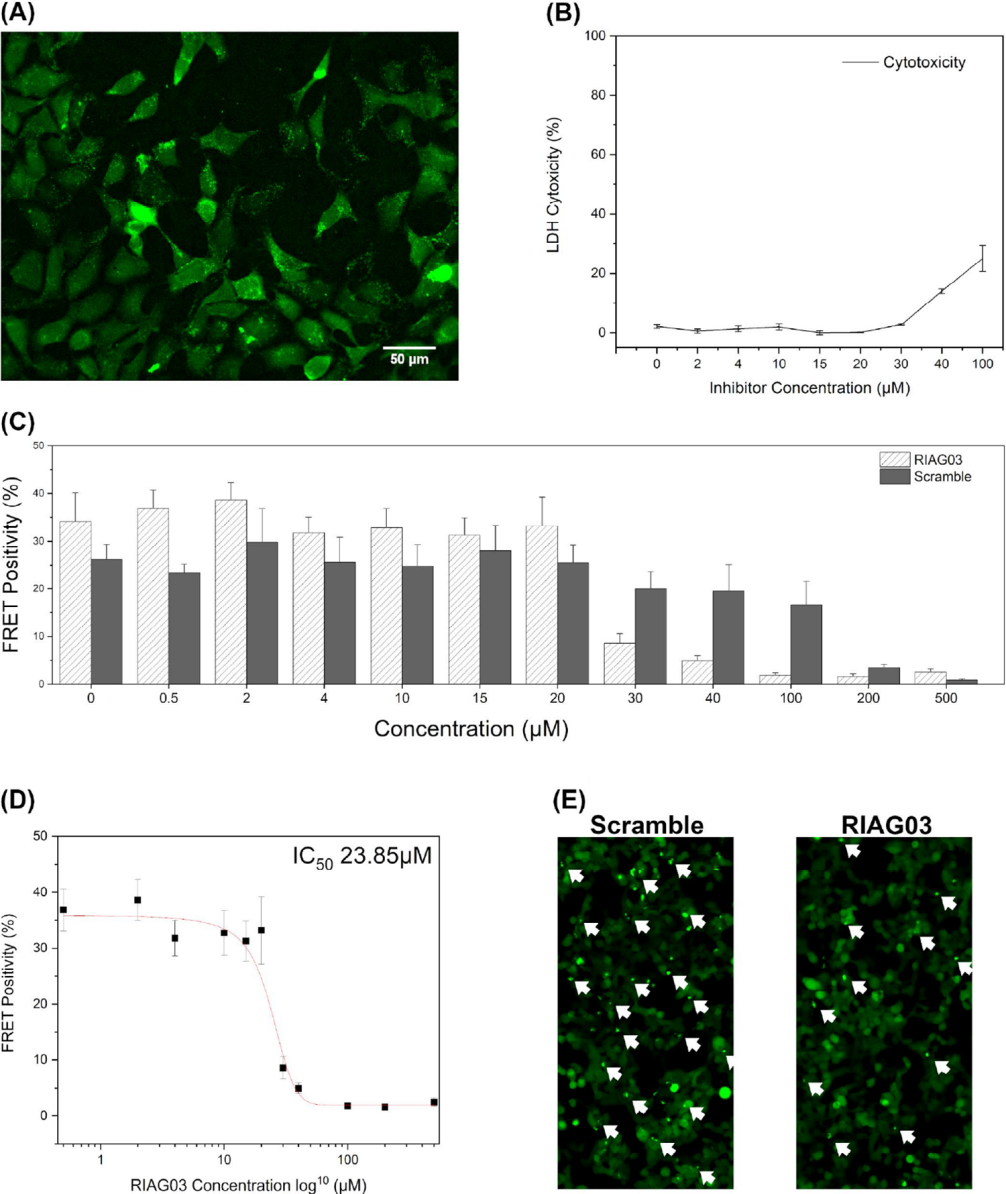
Lead peptide RI‐AG03 penetrates cells and reduces the seeding capacity of Tau seeds. (A) Fluorescence microscopy image depicting cellular uptake of 15 µM 5(6)‐carboxyfluorescein tagged RI‐AG03 [FAM‐RI‐AG03] by HEK‐293 cells incubated in DMEM/10% FBS at 37°C, with 5% CO_2_, after 24 h. Cells were seeded to a 96‐well plate at 20,000 cells/100 µL per well and supplemented with FAM‐RI‐AG03. Cells were visualized on a Nikon Eclipse Ti fluorescent microscope using the FITC filter. (B) LDH cytotoxicity assay using varying concentrations of RI‐AG03 co‐incubated with HEK‐293 cells. LDH Cytotoxicity (%) denotes lysed cells. Cells were seeded to a 96‐well plate at 10,000 cells/100 µL per well. Toxicity begins to increase at 40 µM. Experiments were conducted in triplicate. (C) FRET positivity indicative of Tau aggregation in a HEK‐293 biosensor cell line stably expressing Tau repeat domains with the P301S mutation fused to Cer/Clo. Cells were exposed to preformed Tau^2N4R^ fibril seeds that were preincubated with either RI‐AG03 or Scramble peptide for 16 h before transduction. (D) Seeded aggregation is inhibited by RI‐AG03 at doses between 20 and 100 mM, with the IC_50_ being 23.85 mM. (E) Fewer green puncta indicative of Tau aggregates are seen in representative fluorescence microscopy images of the biosensor cells after treatment with preformed fibrils incubated with 30 µM RI‐AG03 compared with those incubated with Scramble peptide.

To explore the toxicity of RI‐AG03, its effects were studied in cells and in vivo. To have translational potential, RI‐AG03 needs to penetrate cells and demonstrate efficacy in suppressing Tau aggregation in cells without displaying toxic effects at efficacious doses. Using a fluorescently tagged version of the inhibitor, FAM‐RI‐AG03 was visualized by immunofluorescence and confirmed penetration of HEK‐293 cells (Figure 5A). Untreated cells which serve as the controls for this experiment are shown in Figure S12. Furthermore, using a Pierce lactate dehydrogenase cytotoxicity assay, RI‐AG03 was found to be non‐toxic at doses up to 30 µM over 24 h (Figure 5B).

To test RI‐AG03 efficacy in mammalian cells, we utilized a fluorescence resonance energy transfer (FRET) biosensor HEK293 cell line that stably expresses the Tau repeat domain (RD) with the P301S mutation fused to mCer and mClo. These cells^45^ or prior generations^71^ have been used extensively to quantify Tau seeding activity and measure intracellular aggregation.^71^ RI‐AG03 was preincubated in escalating amounts with 5 µM (monomer equivalent) preformed Tau^2N4R^ fibrils for 16 h and fibrils were diluted to 10 nM, sonicated, and combined with Lipofectamine 2000 to facilitate transduction. Lipofectamine complexes were added to the biosensor cell media for 24 h and cells were harvested for flow cytometry. Seeded aggregation was quantified by the percentage of FRET positivity. Inhibition of Tau fibril seeding by RI‐AG03 was observed from 30 µM until 100 µM (Figure 5C), demonstrating that RI‐AG03 can inhibit the seeded aggregation of preformed fibrils in mammalian cells. A decrease in FRET positivity was observed at concentrations of RI‐AG03 and scramble peptide above 100 µM, indicating peptide‐induced toxicity at these higher doses (Figure 5C). The IC_50_ for the inhibition of seeded Tau aggregation by RI‐AG03 in the biosensor cells was estimated at 23.85 µM (Figure 5D). Representative fluorescence microscopy images of the biosensor cells after treatment with preformed fibrils incubated with 30 µM RI‐AG03 or scramble peptide are shown in Figure 5E, with green puncta representing seeded inclusions of TauRD‐P301S that clearly show fewer aggregates in cells treated with the former compared to the latter. These data show that RI‐AG03 reduces Tau seeding in mammalian cells.

To elucidate therapeutic efficacy in an in vivo model, RI‐AG03 was tested in a well‐established *Drosophila* model of Tauopathy wherein neural expression of hTau^2N4R^ leads to hyperphosphorylation,^72^ aggregations,^12^ neurodegeneration,^73^ and multiple AD‐like phenotypes including shortened lifespan and cognitive deficits.^49^ This is an ideal model to test aggregation inhibitors like RI‐AG03 as the phenotypes we have chosen are entirely aggregation dependent.^16^ To assess RI‐AG03 on Tau‐induced neurodegeneration, it was fed to *Drosophila* expressing hTau^2N4R^ in retinal photoreceptors using the eye‐specific GMR‐Gal4 driver. These human‐Tau overexpressing flies undergo profound aggregation‐induced neurodegeneration in the photoreceptors, reflected in a reduced eye size compared to controls. Eye size was significantly improved in hTau^2N4R^ transgenics when treated with 40 µM RI‐AG03 when compared to the untreated hTau^2N4R^ flies (Figure S13).

**FIGURE 6:**
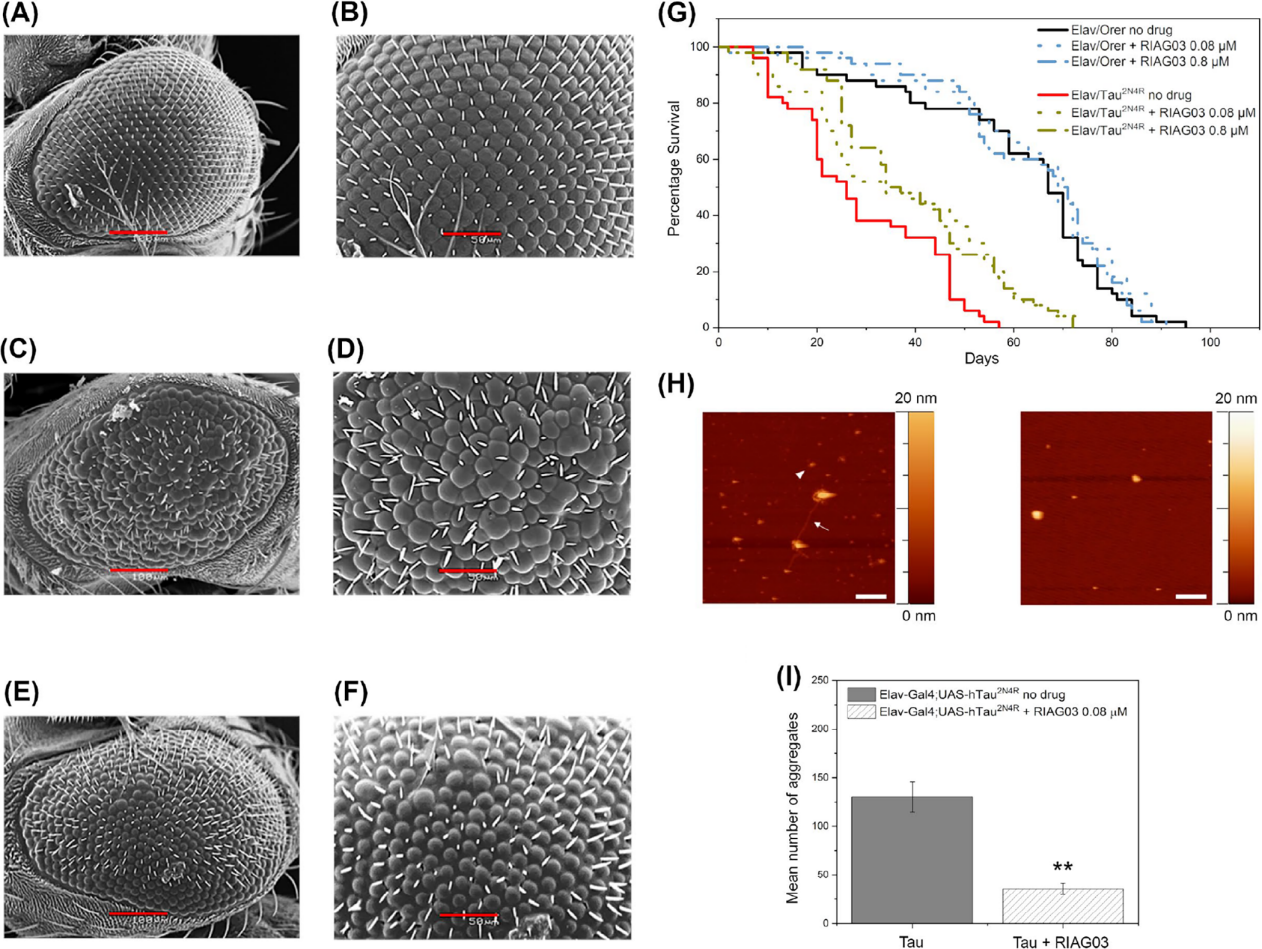
Lead peptide RI‐AG03 reduces tau aggregation and suppresses phenotypes in a model of tauopathy. (A–F) SEM images of the eyes of drosophila melanogaster at 100 (A, C, E) and 50 (B, D, F) microns from left to right; *N* = 6 (A, B) healthy GMR‐GAL4 (C, D) GMR/hTau^2N4R^ flies without treatment (E, F) GMR‐hTau^2N4R^ flies treated with RI‐AG03 20 µM. (G) Survival curves of control (Elav/Orer) and Tau overexpressing flies (Elav/hTau^2N4R^) treated with low (0.08 µM) and high (0.8 µM) doses of the RI‐AG03, respectively, and no treatment. Log‐Rank test *n* = 100 per genotype/treatment group, *p* = 0.0007 and 0.0004, respectively. (H and I) Visualization (H) and quantitation (I) of reduction of Tau aggregates after 0.08 µM inhibitor treatment using atomic force microscopy imaging on an insoluble Tau prep obtained from 6‐week transgenic flies expressing hTau^2N4R^ pan‐neurally. Without RIA03 treatment there is evidence of fibrils (arrow) and oligomers (arrowhead). After treatment, no fibrils are seen, only large spherical structures. *n* = 3, Unpaired *t*‐test *p* = 0.0045.

SEM images of the healthy controls (Figure 6A/B) demonstrated organized eye‐morphology with ordered ommatidia and bristles, whereas the hTau^2N4R^ transgenics displayed fused ommatidia and missing bristles in the anterior portion of the eye leading to the characteristic Tau‐induced “rough‐eye” phenotype (Figure 6C/D). RI‐AG03 treatment partially rescued this “rough‐eye” phenotype by improving the number and morphology of bristles and reducing the number of abnormal ommatidia (Figure 6E/F). Again, scrambled RI‐AG03 peptide did not improve this phenotype (Figures S14/15). The ommatidial disorganization that gives rise to this rough eye phenotypes is now routinely quantified using the “Flynotyper” ImageJ plugin which provides an ommatidial disorderliness index (Odld).^48^ As depicted in Figure S12E, expression of hTau^2N4R^ causes significant ommatidial disorganization which is significantly improved by treatment with RI‐AG03.

To test whether this rescue of Tau‐induced rough‐eye phenotype could be extended to ameliorate aggregation‐induced phenotypes at the organismal level, the impact of low‐dose (0.08 mM) or high‐dose (0.8 mM) RI‐AG03 on the lifespan of flies expressing hTau^2N4R^ pan‐neuronally was assessed. While the lifespan of control flies ranged from 80 to 90 days and was unaltered by inhibitor treatment, the lifespan of the hTau^2N4R^ flies was significantly improved by treatment with both high and low doses of inhibitor (Figure 6G). Median survival of hTau^2N4R^ flies increased from 26 to 35 days (35% improvement) and from 26 to 33 days (27% improvement) following treatment with high and low doses of RI‐AG03 respectively (*p* < 0.0005, *n* = 100). In contrast, survival of controls was not significantly altered by treatment of either dose of inhibitor (67–70; *p* > 0.05, *n* = 100) reinforcing the findings from the cell culture data that RI‐AG03 is non‐toxic at the tested therapeutic doses which inhibit Tau aggregation, even when given to flies for the entire duration of their lifespan.

The suppression of these two Tau‐induced phenotypes, known to be aggregation‐dependent^16^ suggests that RI‐AG03 is efficacious in reducing Tau aggregation in vivo. To provide direct evidence for this and to ascertain if RI‐AG03 was *behaving* in vivo as it did in vitro by preventing the formation of long fibrils and encouraging the formation of amorphous structures instead, AFM was carried out on detergent‐insoluble fractions that enrich for fibrillar Tau using established protocols.^69^ In untreated hTau^2N4R^ flies there was evidence of both oligomeric and fibrillar Tau species (arrowheads and arrows, respectively, in Figure 6E), but in RI‐AG03 treated flies, there were no fibrils, just amorphous structures, akin to GTOs that we have previously described,^9^ some around 40 nM in size, resembling those formed in vitro in the presence of RI‐AG03 (Figure 4C). Visualization (Figure 6H) and quantification (Figure 6I) of the numbers of each of these structures revealed that there was a 73% reduction in both oligomeric and fibrillar species after 6 weeks of treatment with inhibitor, coinciding with the improvement in lifespan. This suppression of Tau aggregate formation is significant (*p* = 0.0045). These data demonstrate that RI‐AG03 acts in the same manner in vitro and in vivo, reducing the formation of toxic Tau aggregates responsible for Tau‐dependent phenotypes, and altering the aggregation pathway to encourage the formation of off‐pathway non‐toxic GTO‐like amorphous species between 30 and 50 nm in diameter.

These data are supported by a previous study of ours in which we described the formation of similarly sized large spherical GTO‐like structures, with reduced β‐sheet content, forming in conditions where Tau‐aggregation dependent phenotypes are rescued through reduced phosphorylation.^69^ RI‐AG03 mediated suppression of these Tau phenotypes supports the concept that it is not necessarily Tau aggregates that are toxic, but aggregates that have β‐sheet heavy amyloidogenic structure. Furthermore, some Tau aggregates, particularly large amorphous oligomers with low β‐sheet structure may be protective, as is the case with those formed in the presence of RI‐AG03 in this study and those formed following suppression of Tau phosphorylation in our previous study.^9^, ^69^

Overall, we have shown that RI‐AG03 is stable and capable of entering cells, crossing the *Drosophila* blood‐brain barrier to reduce aggregation of Tau in cells and in vivo and significantly suppress aggregation‐dependent phenotypes including neurodegeneration and shortened lifespan in a *Drosophila* model of Tauopathy.

## 4 DISCUSSION

We describe the development of a highly specific, stable D‐amino peptide RI‐AG03, for inhibition of Tau aggregation that has displayed properties highly desirable in a disease‐modifying therapeutic for Tauopathies. Designed against the ^306^VQIVYK^311^ aggregate‐promoting region of Tau, RI‐AG03 is unique in that it also interacts with and suppresses aggregation prompted by the ^275^VQIINK^280^ region. To our knowledge, there are no peptide inhibitors that inhibit aggregation of both ^306^VQIVYK^311^ and ^275^VQIINK^280^ containing Tau species. As such it has potential as a treatment for all Tauopathies.

RI‐AG03 is proteolytically stable and capable of entering cells in vitro and in vivo, crossing the *Drosophila* blood‐brain barrier after oral consumption to reduce Tau aggregation and significantly suppress aggregation‐dependent phenotypes including neurodegeneration and shortened lifespan. It achieves these beneficial effects by specifically interacting with Tau to encourage the formation of off‐pathway amorphous aggregates with reduced β‐sheet content, that are analogous to large non‐amyloidogenic oligomers that have been described in other instances of reduced tau toxicity.^9^, ^69^

Tau aggregation inhibitors have been devised and studied for the past 20 years with several initial reports describing the promising potential of numerous small molecules ranging from amino acid derivatives like CL‐NQTrp,^74^ to dye‐derivatives like cyanine^65^ and methylene blue.^75^ While there is no doubt that all these small molecules employ different mechanisms to effectively reduce Tau aggregation, in vitro and for some also in vivo, their utility as therapeutic agents is limited because their mode of action is often non‐specific so they invariably impact other proteins and cause unwanted side‐effects.^63^ Additionally, some of these small molecules, like cyanine, reportedly both suppress and promote Tau fibril formation, depending on the dose used,^76^ possibly by encouraging the formation of pre‐fibrillar oligomeric structures, as has been reported for methylene blue.^77^ This may explain why many small molecule aggregation inhibitors, have not translated into efficacious therapies, and those like methylene blue, that have been tried in phase III clinical trials have failed. RI‐AG03 also appears to encourage the formation of amorphous aggregates at the expense of Tau fibrils, but unlike the oligomers formed in the presence of small molecules like methylene blue,^66^, ^77^ the RI‐AG03 induced aggregates do not seem to be toxic. We hypothesize that this is possible because RI‐AG03 promotes off‐pathway aggregation of Tau, folding it into large amorphous aggregates that have reduced β‐sheet content.

We designed RI‐AG03 because, in recent years, interest in Tau aggregation inhibitors has shifted to small peptides, that are considered to be superior alternatives to small molecules. A key reason for this is that they can be created specifically to target identified regions found only in the Tau protein and are therefore less likely to have any off‐target toxic effects.^78^ Indeed, RI‐AG03 has no adverse effect in vivo in our *Drosophila* model where there was no difference in the longevity of control flies fed diluent alone or RI‐AG03 for their entire lifespan (over 95 days). Though designed against the ^306^VQIVYK^311^ aggregate‐promoting hotspot of Tau, RI‐AG03 interacts with and suppresses aggregation prompted by this and the ^275^VQIINK^280^ hotspot. This is significant as the latter is believed to be the more potent driver of aggregation, which is why many other peptide inhibitors are designed to inhibit aggregation driven by ^275^VQIINK^280^ alone.^24^, ^26^ Others have also designed peptide inhibitors that appear to target the ^306^VQIVYK^311^ region,^25^, ^74^ like RI‐AG03, but to our knowledge there are no peptide inhibitors that inhibit aggregation of both ^306^VQIVYK^311^ and ^275^VQIINK^280^ containing Tau species. Therefore, existing peptide inhibitors have been found to be effective in reducing aggregation of only specific Tau species, fragments, or Tau^2N4R^ isoforms that contain either of these aggregation hotspots.^26^ RI‐AG03 is unique in that regard as it suppresses aggregation of multiple Tau species, containing both ^306^VQIVYK^311^ and ^275^VQIINK^280^ hotspots. This should make RI‐AG03 efficacious for the treatment of all Tauopathies whether the aggregating species has only ^306^VQIVYK^311^ containing Tau aggregates (3R Tauopathies) or both ^306^VQIVYK^311^ and ^275^VQIINK^280^ containing Tau aggregates (e.g., AD and 4R Tauopathies).

Being a D‐amino acid peptide, RI‐AG03 is proteolytically very stable, and as evidenced by our *Drosophila* data, it crosses the blood‐brain barrier after oral consumption to reduce Tau aggregation in the brain. Crucially, RI‐AG03's inhibitory effect is not dependent upon interaction with the aggregation inducer, as is reported for other peptide inhibitors,^79^ which is an essential requirement for all potential Tau‐aggregation inhibitors as the aggregation inducer in vivo in sporadic Tauopathies is unknown so the aggregation inhibitor should be independent of the aggregation inducer.

Collectively our data describe several properties and attributes of RI‐AG03 that make it worthy of further exploration as a disease‐modifying candidate for reducing pathogenic Tau aggregation in Tauopathies. Given the real interest in reducing Tau aggregation and the potential clinical benefit of using such agents in clinical practice, the therapeutic potential of RI‐AG03 should be explored further in future studies in rodent models of Tauopathy.

## CONFLICT OF INTEREST STATEMENT

The authors declare that they have no conflict of interest. Author disclosures are available in the supporting information.

## CONSENT STATEMENT

The data analyzed in this research were collected from non‐human sources. Therefore, relevant ethical approval and informed consent were not required as no human subjects were recruited.

## Supporting information



Supporting Information

Supporting Information
