# Supplementary material for: A novel peptide‐based tau aggregation inhibitor as a potential therapeutic for Alzheimer's disease and other tauopathies
Source: Alzheimers Dement. 2024 Oct 3;20(11):7788–804. doi: 10.1002/alz.14246 (PMC11567856; doi:10.1002/alz.14246)
Supplement: Supplementary file 1 — Supporting Information [file ALZ-20-7788-s002.pdf]

A novel peptide-based Tau aggregation inhibitor as a potential therapeutic for Alzheimer's disease and other Tauopathies

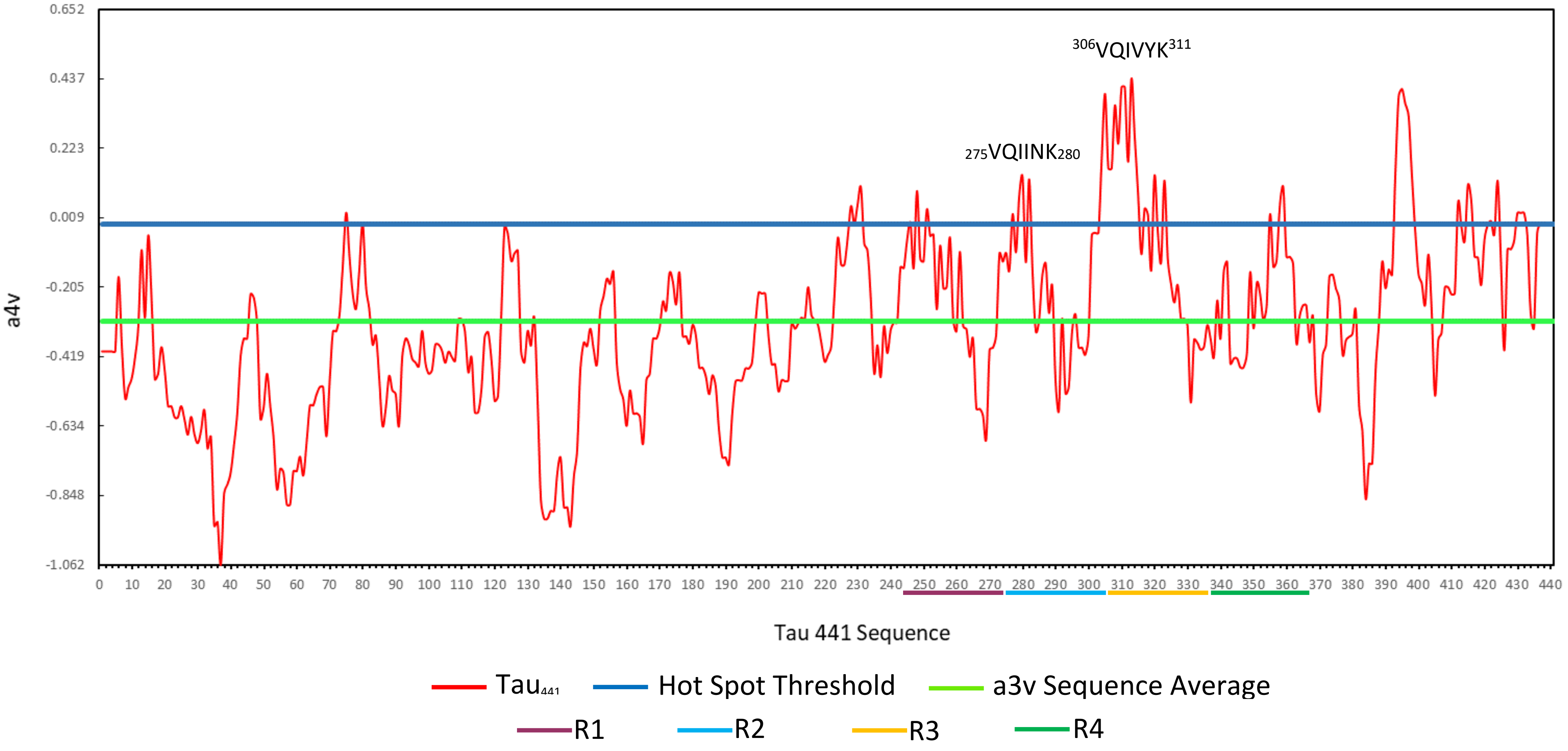

Figure S1: AGGRESCAEN average aggregation propensity for Tau_{441} residues. VQIVYK exceeds the hot spot threshold (blue line) significantly. R1-4 signifies the repeat regions in Tau. Hexapeptides exceeding the hot spot threshold are highlighted as residues likely to aggregate.

A novel peptide-based Tau aggregation inhibitor as a potential therapeutic for Alzheimer's disease and other Tauopathies

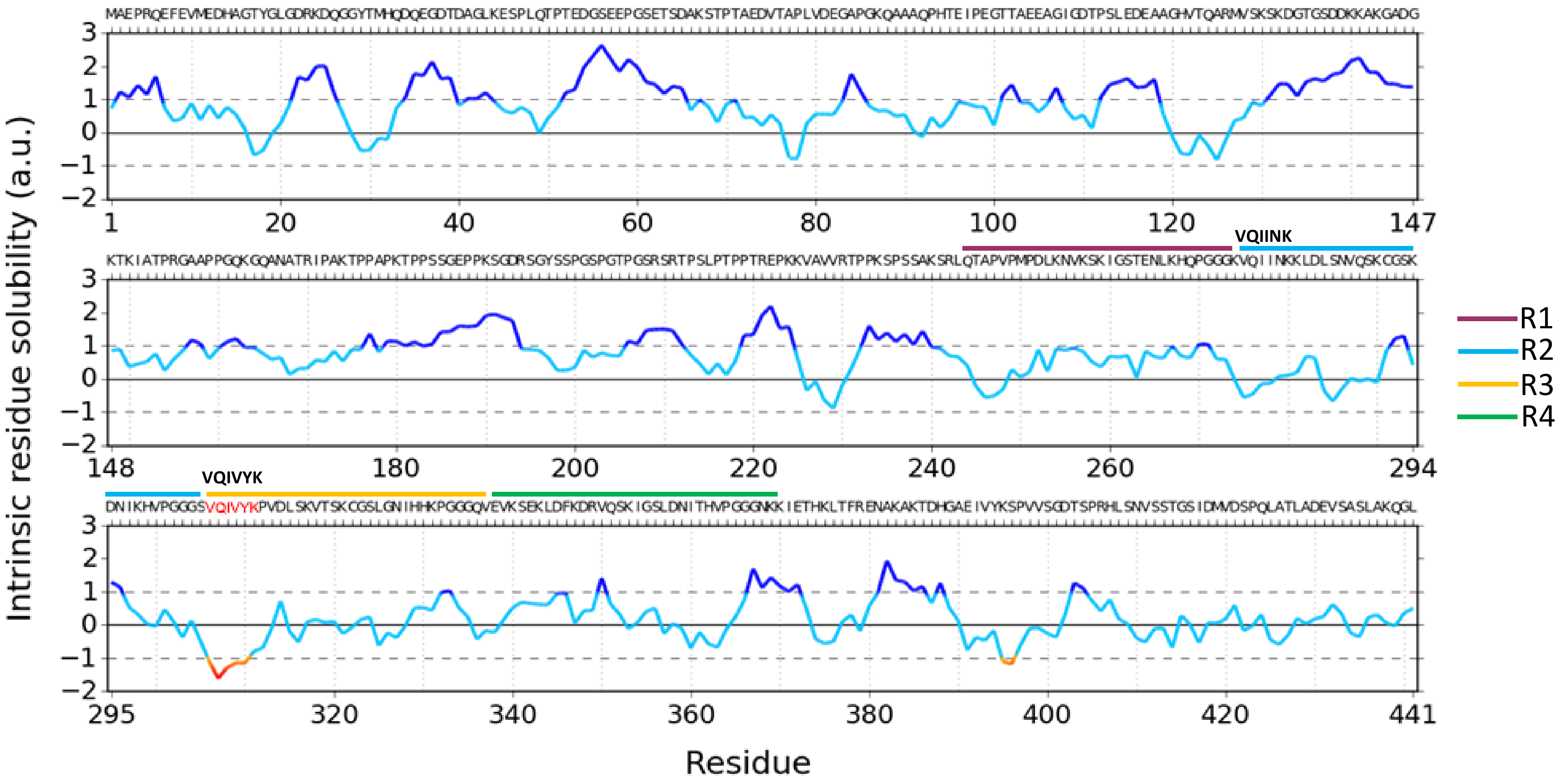

Figure S2: CamSol Intrinsic residue solubility for Tau$_{441}$ residues. VQIVYK contains hydrophobic residues valine and isoleucine. The hexapeptide is identified with a red line, suggesting an aggregation hot spot. Blue lines suggest soluble regions. R1-4 signifies the repeat regions in Tau.

A novel peptide-based Tau aggregation inhibitor as a potential therapeutic for Alzheimer's disease and other Tauopathies

(A).

**VQI(x)YK** **Above aggregation threshold ✗**											
Residue substitution	A	C	G	I	L	M	F	S	T	W	Y
Aggregation propensity value (Na4vSS)	23.3	36.1	13.4	60.5	51.7	42.3	59.1	18.2	20.9	44.8	47.2

**VQIVYK**
**55.9**

(B).

**VQI(x)YK** **Below aggregation threshold ✓**								
Residue substitution	R	N	D	Q	E	H	K	P
Aggregation propensity value (Na4vSS)	-0.7	-2.0	-12.7	-0.6	-4.2	3.4	**5.4**	17.4

**VQIVYK**
**55.9**

(C).

**VQ(x)VYK** **Above aggregation threshold ✗**											
Residue substitution	A	C	G	V	L	M	F	S	T	W	Y
Aggregation propensity value (Na4vSS)	18.8	31.6	8.8	51.4	47.1	37.7	54.6	13.6	16.3	40.2	42.7

**VQIVYK**
**55.9**

(D).

**VQ(x)VYK** **Below aggregation threshold ✓**								
Residue substitution	R	N	D	Q	E	H	K	P
Aggregation propensity value (Na4vSS)	-5.3	-6.5	-17.2	-5.1	-8.7	-1.2	**0.9**	12.8

**VQIVYK**
**55.9**

Table S3: Summarises the average aggregation propensity values for VQI(x)YK and VQ(x)VYK sequences, using replacements at (x) with different amino acids for comparison. **A+C:** The conformations which were predicted to aggregate, **B+D:** The sequences which were predicted not to aggregate. Replacement at x with R, N, D, Q, E, H, K and P massively reduced the average aggregation propensity. Residues coloured in red, blue and black are hydrophobic, hydrophilic or neutral, respectively.

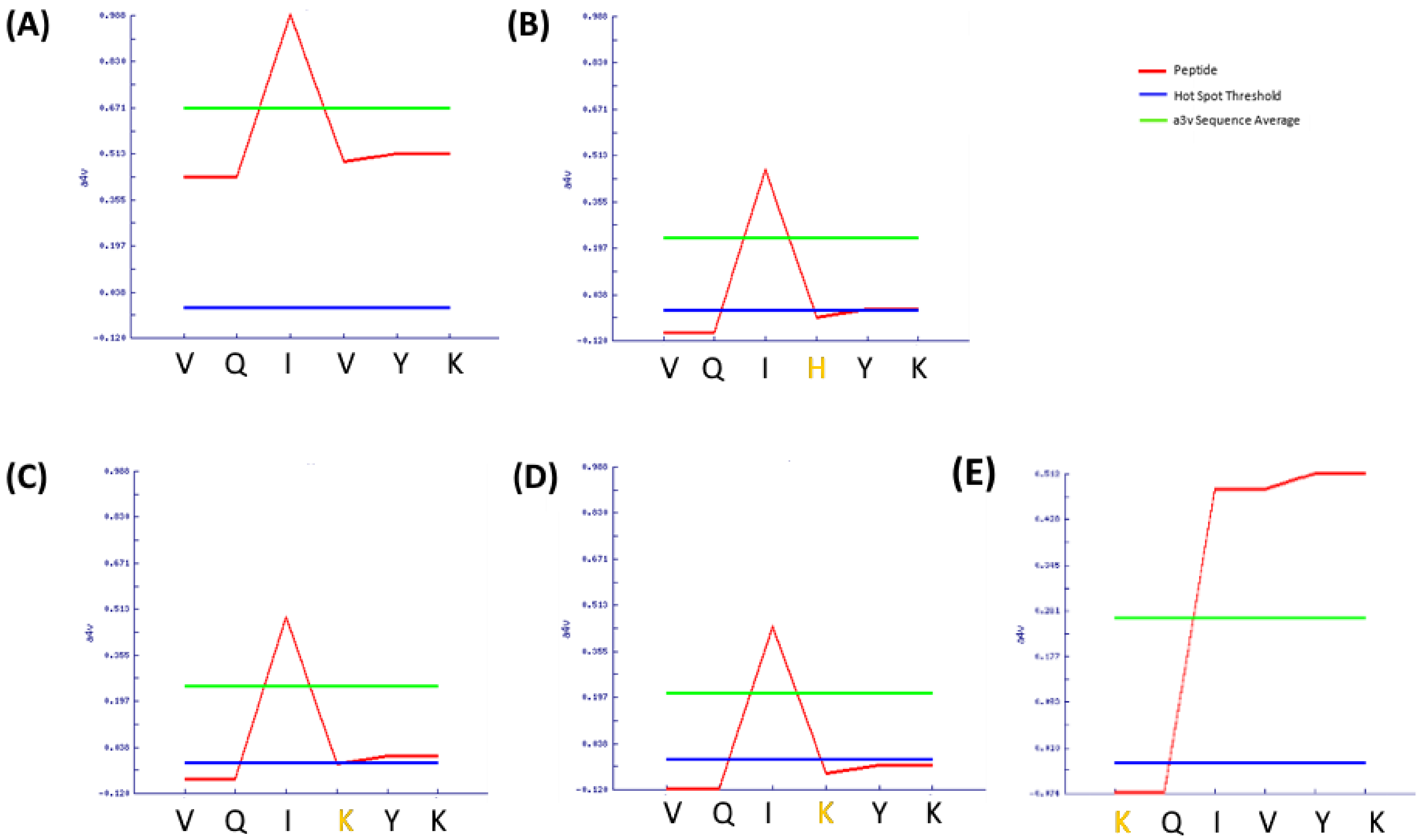

Figure S4: AGGRESCAN average aggregation propensity for Tau residues 301-315 with a replaced amino acid indicated in orange. **a:** Native VQIVYK; **b:** VQI**H**YK; **c:** VQI**K**YK; **d:** VQ**K**VYK; **e**: KQIVYK. Replacing isoleucine or valine greatly reduces the aggregation propensity.

A novel peptide-based Tau aggregation inhibitor as a potential therapeutic for Alzheimer's disease and other Tauopathies

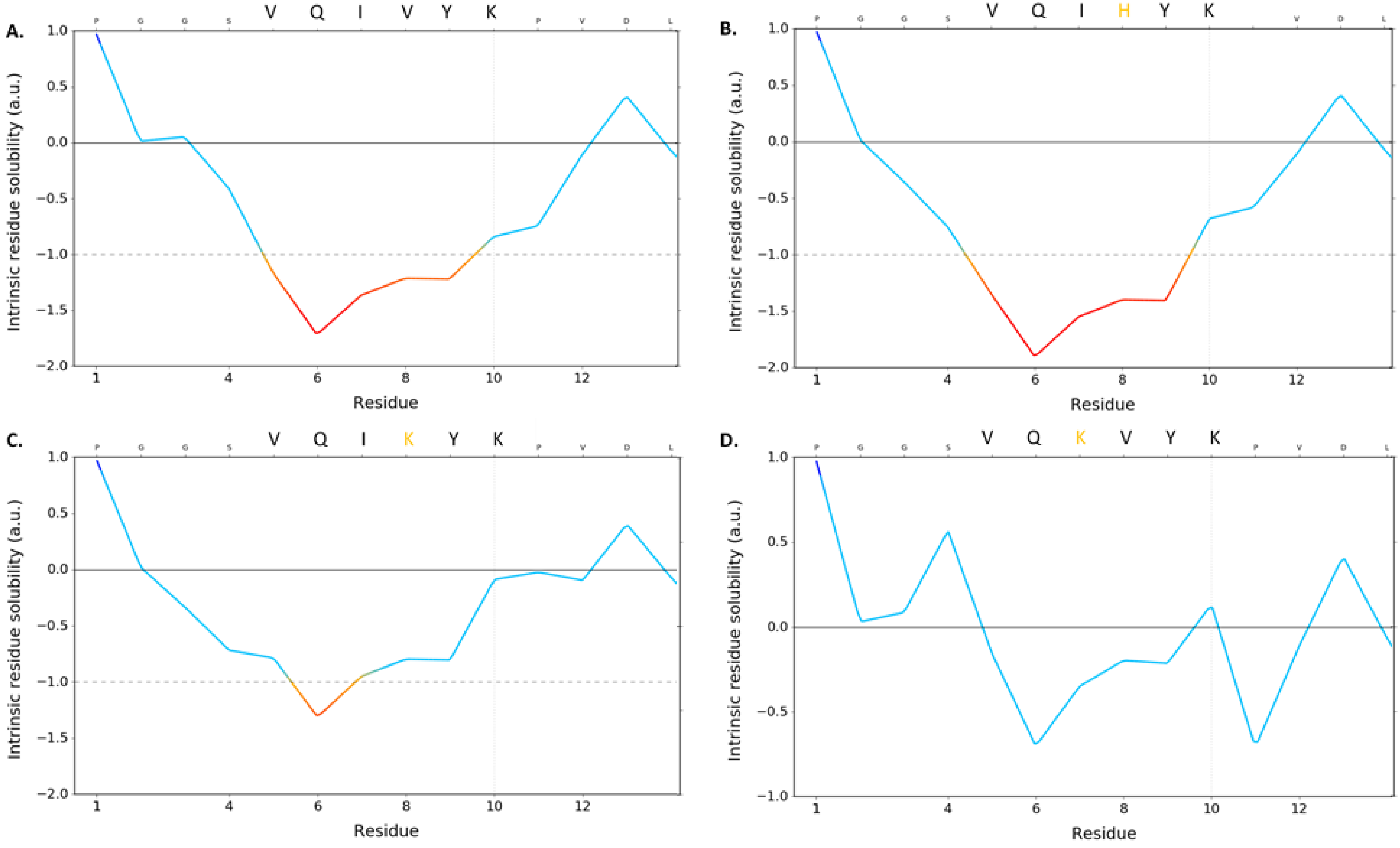

Figure S5: CamSol Intrinsic residue solubility for Tau residues 301-315 with a replaced amino acid indicated in orange. **A:** Native VQIVYK; **B:** VQI**H**YK; **C:** VQI**K**YK; **D:** VQ**K**VYK. Isoleucine appears to be more important for influencing intrinsic solubility than valine is in the VQIVYK sequence.

A novel peptide-based Tau aggregation inhibitor as a potential therapeutic for Alzheimer's disease and other Tauopathies

		VQIVYK ligand			VQIK(Ac)YKP ligand		
**Structure**	**Receptor**	**Orientation**	**Best ICM score**	**H. bonds**	**Orientation**	**Best ICM score**	**H. bonds**
**PHF**	VQIVYK	Parallel	-16.36	4	Anti-Parallel	-28.23	7
**Snake**	VQIVYK	Parallel	-17.55	7	Anti-Parallel	-20.19	11
**Snake**	VQIINK	Parallel	-22.11	8	Parallel	-17.45	10
**Twister**	VQIVYK	Parallel	-32.14	8	Anti/Parallel	-22.95	9
**Jagged**	VQIVYK	Parallel	-21.87	7	Parallel	-25.48	10
	VQIINK	Parallel	-17.29	7	-	-	-

Table S6: Summarises the strongest energy, orientation and hydrogen bond count for VQIVYK and VQIK(Ac)YKP docked to PDB: 5o3l PHF, 6QJH snake filament, 6QJM twister filament and 6QJP jagged filament. The strongest energy binds are highlighted in green.

A novel peptide-based Tau aggregation inhibitor as a potential therapeutic for Alzheimer's disease and other Tauopathies

Peptide ID	Sequence	Purity %
AG01	Ac - R G V Q I I N K G R - NH_2	>90
AG02	Ac - R G V Q I V Y K G R - NH_2	>90
AG02R4	Ac - R R G V Q I V Y K G R R - NH_2	>90
AG02R5	Ac - R G V Q I V Y K G R R R R - NH_2	>90
AGR502	Ac - R R R R G V Q I V Y K G R -N H_2	>90
AG02PR5	Ac - R G V Q I V Y K P G R R R R - NH_2	>90
AG02R6	Ac - R R R G V Q I V Y K G R R R - NH_2	>90
AG02R9	Ac - R G V Q I V Y K G R R R R R R R R - NH_2	>90
AG02TAT	Ac - R G V Q I V Y K G R Y G R K K R R Q R R R - NH_2	>90
AG02ΔI	Ac - R G V Q K(Ac) V Y K G R - NH_2	>90
AG02ΔV	Ac - R G V Q I K(Ac) Y K G R - NH_2	>90
AG03	Ac - R G V Q I K(Ac) Y K P G R R R R R R R R - NH_2	>95
AG03-C	Ac - R G V Q I K(Ac) Y K P G R R R R R R R R C - OH	>95
Scramble AG03	Ac – R G Q P K I K(Ac) Y V G R R R R R R R R - NH_2	>95
AG03M	Ac - R G V(m) Q I(m) K(Ac) Y(m) K P(m) G R R R R R R R R - NH_2	>95
RI-AG03	Ac - r r r r r r r r G p k y k(ac) i q v G r - NH_2	>95
FAM-RI-AG03	Ac - k(FAM) r r r r r r r r G p k y k(ac) i q v G r - NH_2	>95
Poly-R	R R R R R R R R R - NH_2	>95
TAT	Ac - Y G R K K R R Q R R R - NH_2	>95

Table S7: These are the experimental peptide inhibitor designs tested in this research. Underlined portions indicate the predicted Tau binding regions. Peptides were synthesised by Peptide Synthetics (Fareham, UK) and Severn Biotech (Kidderminster, UK). Purity was determined by HPLC-MS. Flanking the peptide binding sequence with RG and GR spacers may improve binding ability by distancing other amino acids, e.g. cell penetrating sequences which could potentially interfere. Activity was enhanced by acetylating and amidating the peptide terminals. Stability of AG03 was enhanced with either N-methylation or retro-inversion.

A novel peptide-based Tau aggregation inhibitor as a potential therapeutic for Alzheimer's disease and other Tauopathies

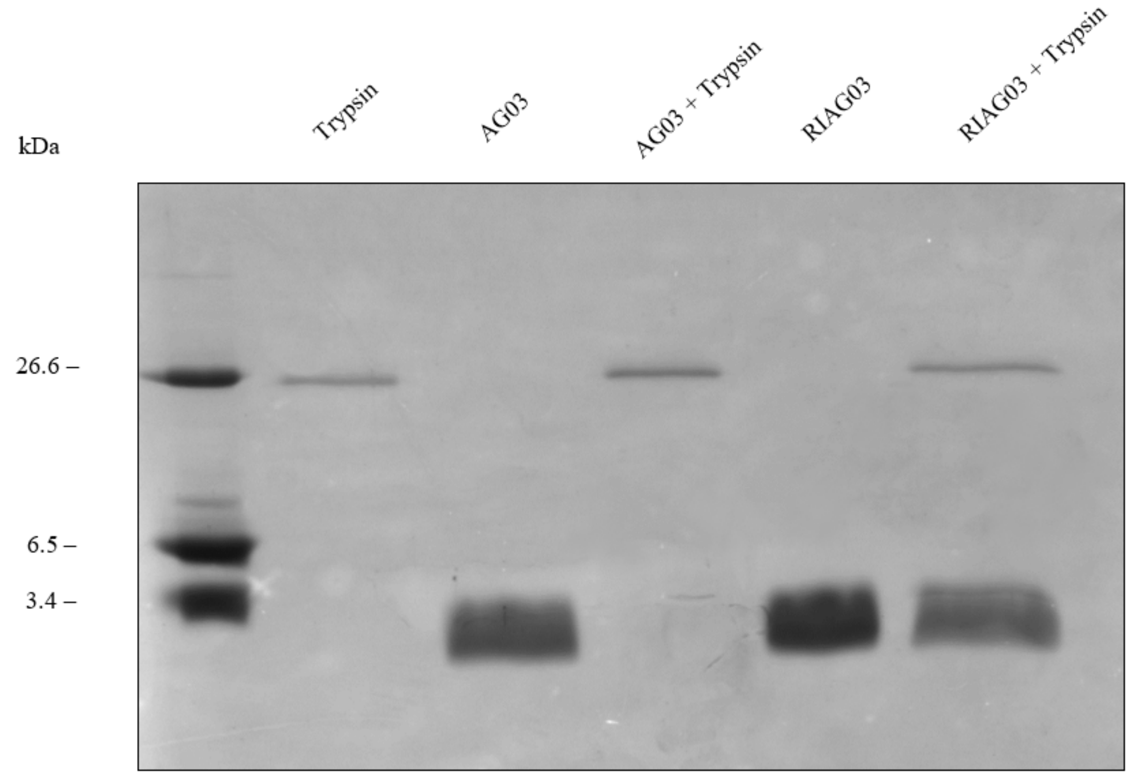

Figure S8: **RI-AG03 resists Trypsin proteolysis.**
15% SDS PAGE gels of AG03 (100 µM) and RI-AG03 (100 µM) treated with and without equimolar Trypsin concentration for 24-hours at 37˚C. AG03 has no signal in the presence of Trypsin whereas RI-AG03 has a strong signal in the presence of Trypsin. Densitometric analysis suggested a 19% reduction in signal after equimolar incubation of RI-AG03 with Trypsin.

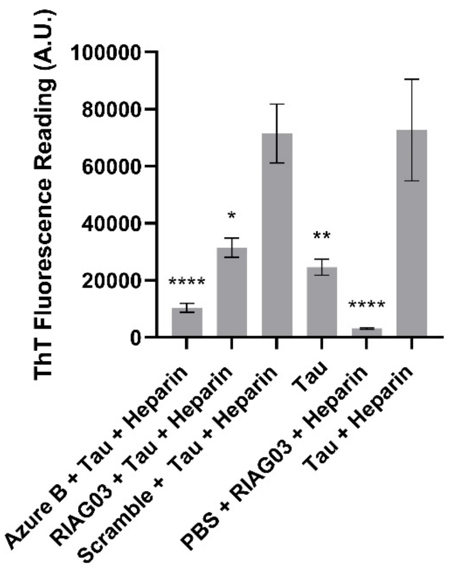

Figure S9: RI-AG03 does not self-associate into β-sheets within 9 days of Tau2N4R aggregation. End-point measurements using ThT fluorescence after 216 hr (9 days) of 2N4R Tau aggregation in the presence of PBS buffer, 1 mM DTT, 20 µM ThT, and 10 µM heparin (pH 7.4) supplemented with inhibitors 20 µM (Azure B, RI-AG03 or RI-AG03 scramble), n=3, One factor repeated measures ANOVA + Tukey post hoc statistical analysis: $P > 0.05$, * $P \leq 0.05$, ** $P \leq 0.01$, *** $P \leq 0.001$.

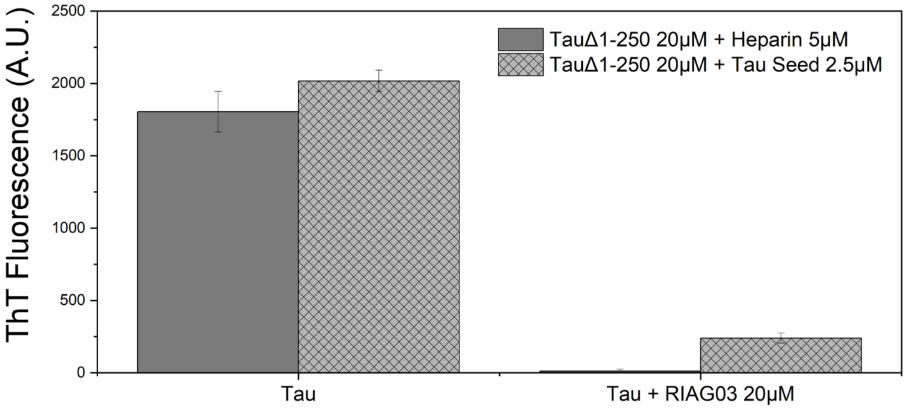

Figure S10: End-point aggregation kinetics of TauΔ1-250 (20μM) in the presence of heparin or Tau seeds and treated with equimolar RI-AG03.

A novel peptide-based Tau aggregation inhibitor as a potential therapeutic for Alzheimer's disease and other Tauopathies

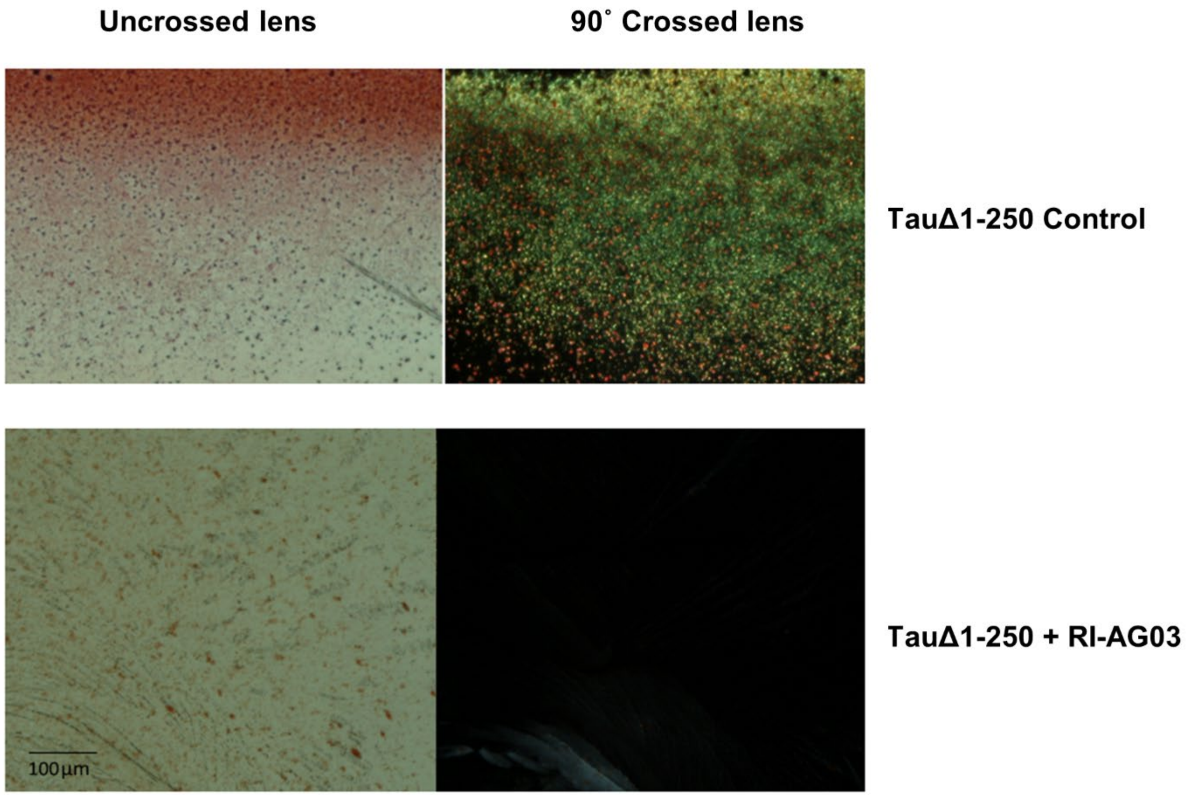

Figure S11: Birefringence data using Zeiss axioscope A1 microscope. $Tau^{\Delta 1\text{-}250}$ with and without presence of RI-AG03, stained with congo red and visualised under uncrossed lens and crossed polarising (90°) lens. The apple-green birefringence, observed for the $Tau^{\Delta 1\text{-}250}$ fibrils is indicative of β-sheet content, and its absence when the aggregates are formed in presence of RI-AG03 suggests that there is profoundly less β-sheet content in the latter.

A novel peptide-based Tau aggregation inhibitor as a potential therapeutic for Alzheimer’s disease and other Tauopathies

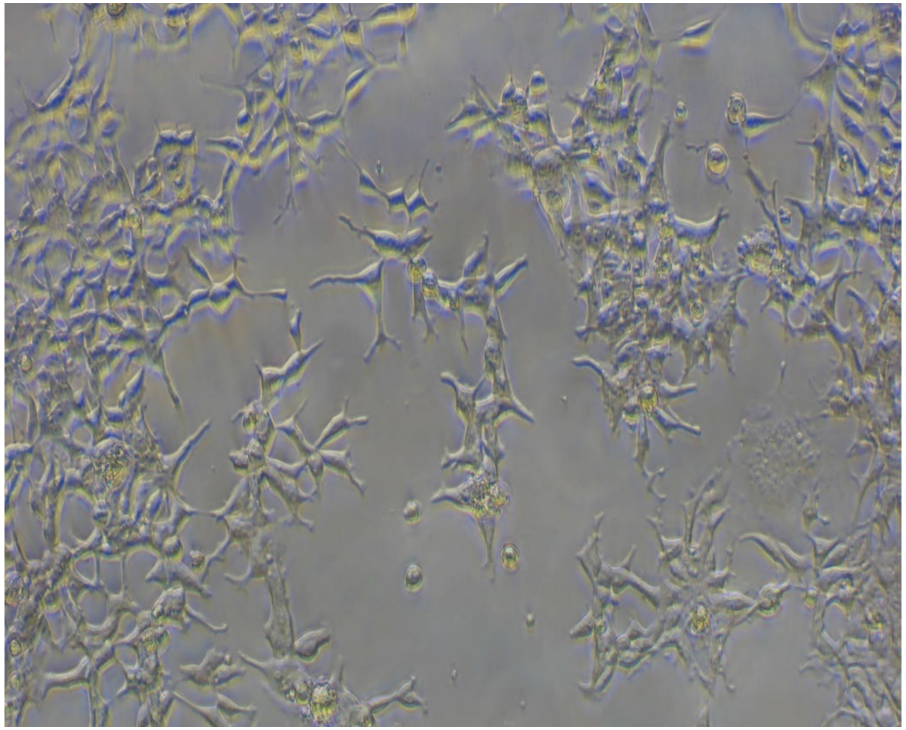

Figure S12: Untreated HEK-293 cells incubated with DMEM/10% FBS at 37°C with 5% CO_2 over 24h. Cells were seeded to a 96 well plate at 20,000 cells/100ul per well and imaged in Leica Phase contrast microscope under 10x magnification.

A novel peptide-based Tau aggregation inhibitor as a potential therapeutic for Alzheimer's disease and other Tauopathies

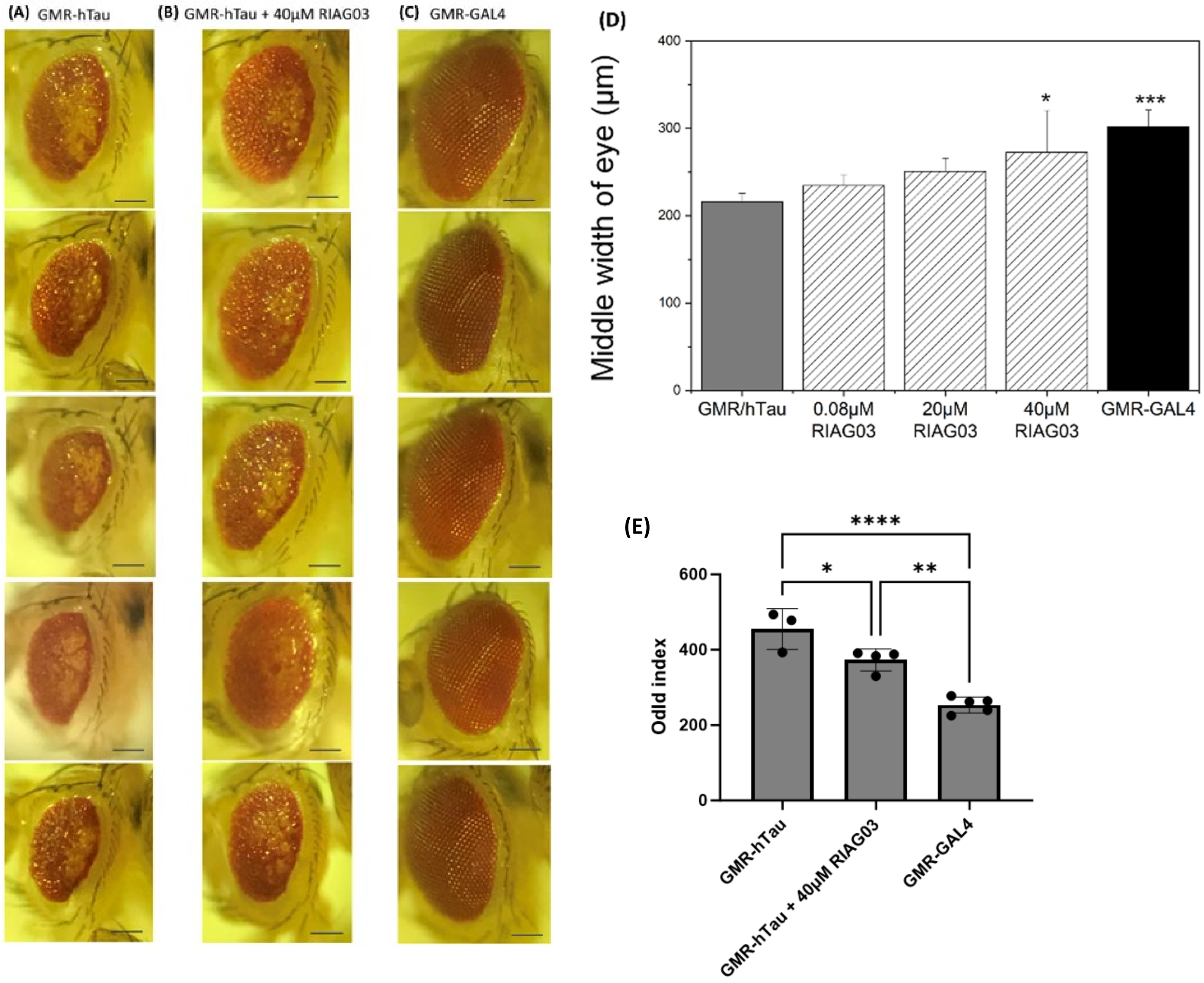

A novel peptide-based Tau aggregation inhibitor as a potential therapeutic for Alzheimer's disease and other Tauopathies

Figure S13: **RI-AG03 treatment partially rescues *GMR*-hTau$_{2N4R}$ rough eye phenotype.**

Shows experimental data of *Drosophila* over-expressing Tau in the eye (GMR-hTau) treated with various concentrations of RI-AG03 and healthy *Drosophila* control (GMR-GAL4).

A-C) Light microscopy images of *Drosophila* eyes expressing Tau (A), treated with RI-AG03 (B), compared to no Tau expressed controls (C). N=5. Error bar = 100µm. Notice the increased eye width in RI-AG03 treated flies, more closely resembling the GMR-GAL4 controls.

D) Dose-dependent increase in eye width with increasing concentrations of RI-AG03 up to 40µM. Data presented as means (n=5/condition) and standard deviation. One factor repeated measures ANOVA + Tukey post hoc statistical analysis was conducted.

E) Phenotypic analysis using the Flynotyper ImageJ plugin (Iyer et al., 2016). Whilst images taken using a light microscope show an obvious restoration of eye size after RIAG03 treatment, we utilized the established Flynotyper ImageJ plugin (Iyer et al., 2016) to calculate the phenotypic score of the eye which better represents measurements contributing toward the rough eye phenotype (REP). This score is based on several measurements of the disorderliness in the ommatidial arrangement of the fly eye and provides an unbiased quantification approach for the REP rescued by RIAG03-treated hTau-expressing flies. The higher the phenotypic score, the more severe the retinal disorderliness. As depicted in Fig E, expression of hTau leads to significant ommatidial disorderliness (as measured by the total distance ommatidial disorderliness index of all stable ommatidia [Odld]) which is significantly improved after RI-AG03 treatment. Data presented as means (n=4-5/condition) and standard deviation. One-way ANOVA with Tukey's post hoc multiple comparisons test was conducted. Adjusted P values *p=0.0270, **p=0.0013, ****p<0.0001.

A novel peptide-based Tau aggregation inhibitor as a potential therapeutic for Alzheimer's disease and other Tauopathies

(A).

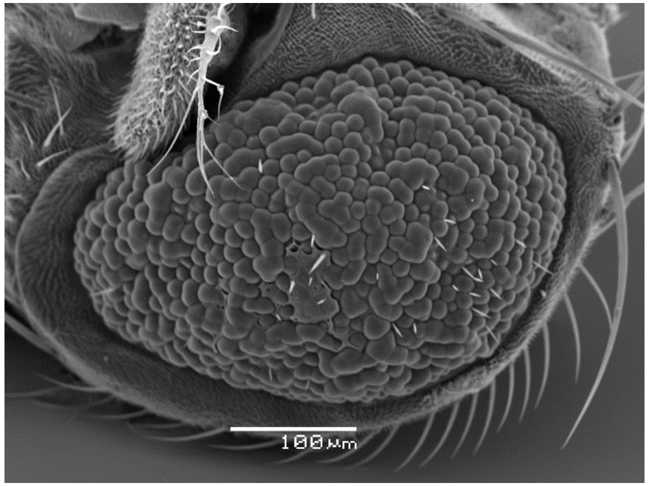

(B).

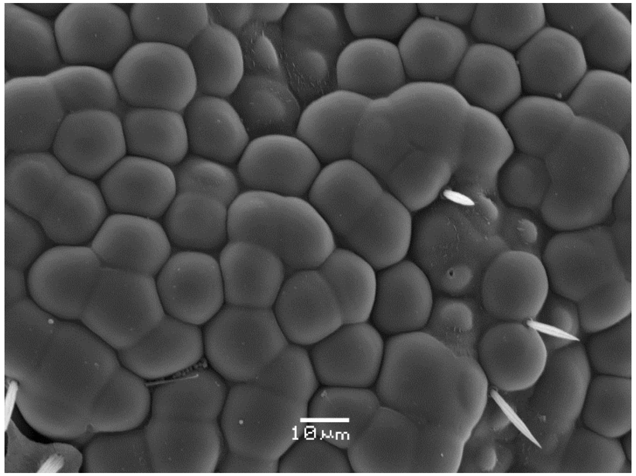

(C).

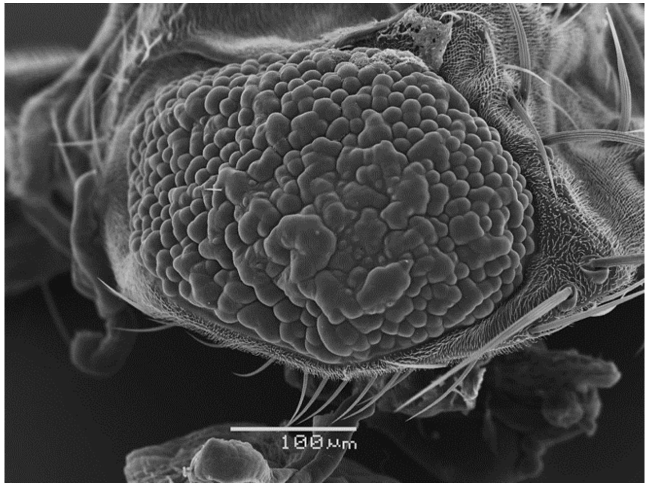

(D).

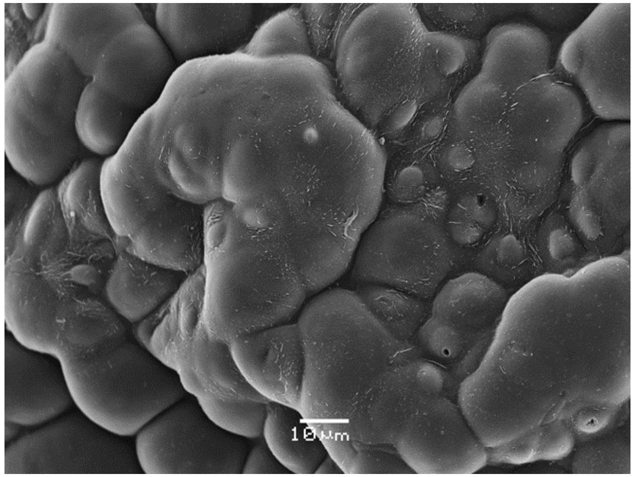

Figure S14: **RI-AG03 inhibitor treatment partially rescues rough eye phenotype. Scramble peptide controls retain phenotype.**

SEM images of the eyes of male flies (T0-T1) treated with 20µM RI-AG03 **(A-B)** and flies treated with a scramble peptide **(C-D).** Note the inhibitor eyes have smaller more normal looking ommatidia with fewer fused ommatidia than the scramble eyes. Although the inhibitor eyes were not normal in appearance, they were closer to the appearance of WT eyes both in size and shape than the scramble eyes. N=6.

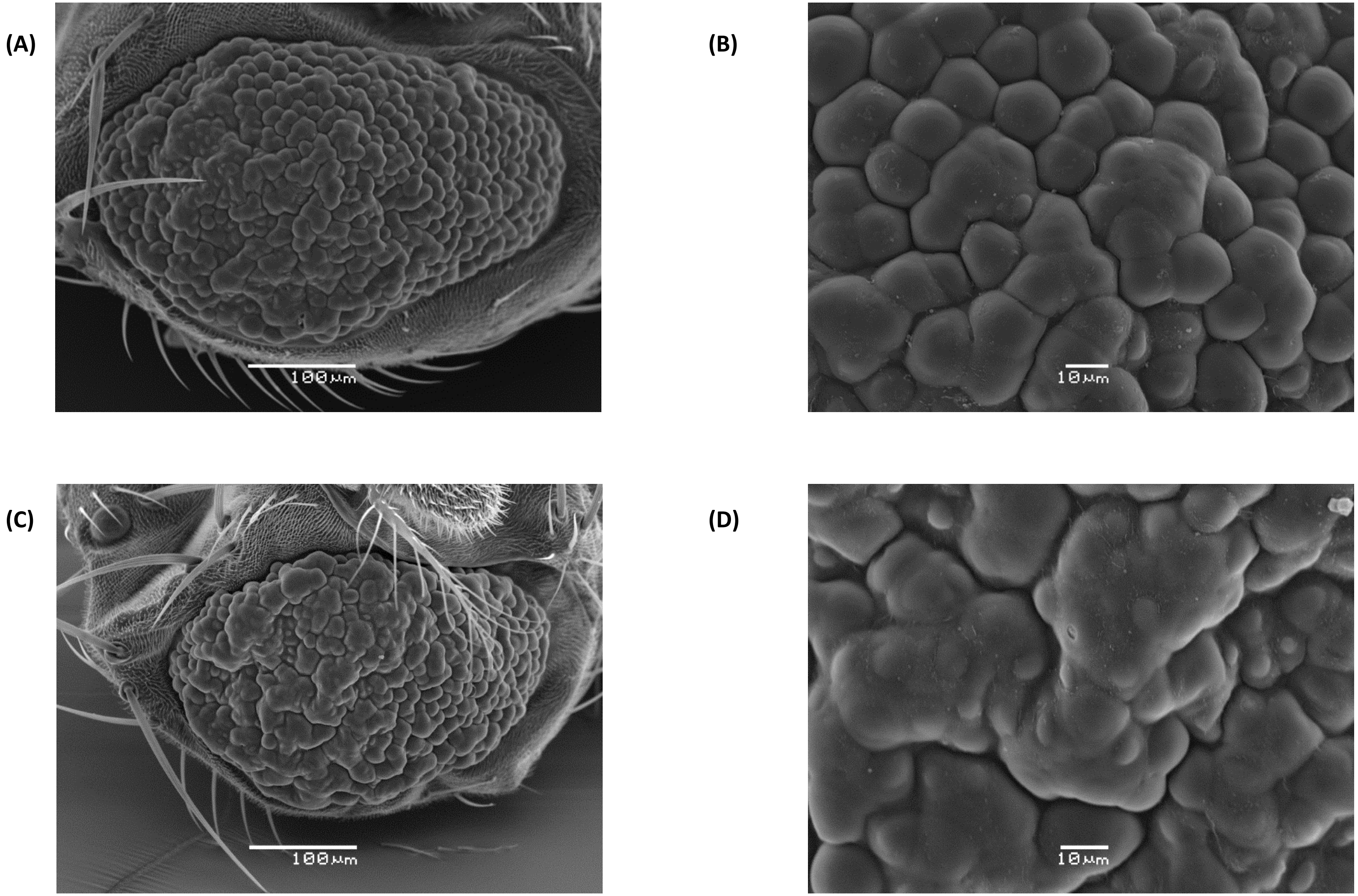

Figure S15: **RIA-G03 inhibitor treatment partially rescues rough eye phenotype. Scramble peptide controls retain phenotype.**

SEM images of the eyes of female flies (T0-T1) treated with 20µM RIA-G03 **(A-B)** and flies treated with a scramble peptide **(C-D).** Note the inhibitor eyes have smaller more normal looking ommatidia with fewer fused ommatidia than the scramble eyes. Although the inhibitor eyes were not normal in appearance, they were closer to the appearance of WT eyes both in size and shape than the scramble eyes. N=6.
